# Correlation between cholesterol and ambulatory blood pressure in postmenopausal osteoporotic patients: a *post-hoc* analysis of an observational study

**DOI:** 10.3389/fmed.2025.1662973

**Published:** 2025-12-08

**Authors:** Zebin Lin, Tianlong Wu, Yipin Zhao, Chizhen Wang, Gaofeng Wu

**Affiliations:** 1 Department of Geriatrics, Xiamen Institute of Geriatric Rehabilitation, Zhongshan Hospital Affiliated to Xiamen University, Xiamen, China; 2 Department of Cardiology, QingPu Branch of Zhongshan Hospital Affiliated to Fudan University, Shanghai, China; 3 Department of Cardiology, Fuwai Central China Cardiovascular Hospital, Zhengzhou, China

**Keywords:** ambulatory blood pressure, hypertension, osteoporosis, cholesterol, postmenopausal women

## Abstract

**Objective:**

To explore the correlation between cholesterol levels and 24-h dynamic blood pressure in postmenopausal women with osteoporosis.

**Methods:**

Ambulatory blood pressure monitoring was performed using a portable automated device, and cholesterol levels were measured using a fully automated biochemical analyzer. The relationship between cholesterol and blood pressure was analyzed using Shepherd’s Pi correlation and generalized additive models (GAM) with piecewise linear regression to identify threshold effects.

**Results:**

The mean age of the participants was 73 years. Shepherd’s Pi correlation analysis revealed a negative correlation between total cholesterol (TC) and intact parathyroid hormone (iPTH), as well as between high-density lipoprotein cholesterol (HDL-C) and *β*-crosslaps. Using GAM and piecewise linear regression to identify threshold effects, we found that when TC was ≥5.5 mmol/L, it was significantly and positively associated with 24-h mean systolic blood pressure (SBP) (*β* = 12.62, 95% CI: 5.23–20.00, *p* < 0.001), as well as with diurnal and nocturnal mean SBP. Similarly, low-density lipoprotein cholesterol (LDL-C) ≥ 3.9 mmol/L showed a positive correlation with multiple blood pressure parameters, including 24-mSBP (*β* = 18.33, 95% CI: 5.93–30.74, *p* = 0.004), diastolic blood pressure (DBP), and both diurnal and nocturnal measurements. No significant associations were observed below these threshold values. In contrast, HDL-C < 1 mmol/L was negatively correlated with 24-mSBP (*β* = −32.13, 95% CI: −57.67 to −6.60, *p* = 0.014), but not with DBP.

**Conclusion:**

A threshold effect exists between cholesterol and blood pressure in postmenopausal osteoporosis patients. Elevated TC and LDL-C beyond specific thresholds, as well as low HDL-C, are associated with higher blood pressure levels.

## Introduction

Hypertension is a common chronic disease worldwide, and its incidence is increasing year by year. There are many factors contributing to hypertension, including salt intake, alcohol consumption, smoking, etc. (1). Furthermore, studies have shown that there is also a correlation between blood lipids and hypertension (2).

Menopause is a necessary physiological stage for all women. During this period, due to the decrease in estrogen production, many pathological outcomes and increased disease risks occur. It is more likely to cause conditions such as osteoporosis, atherosclerosis and hypertension (3). In a study, after adjusting for age and body mass index, the prevalence of hypertension among postmenopausal women was twice that of premenopausal women (4). In the NHANES III study, the incidence of hypertension was compared among different genders. Specifically, among patients over the age of 59, the incidence rate of females was higher than that of males (5). The lack of estrogen in postmenopausal women leads to an increase in blood pressure (6). The deficiency of estrogen is also a major cause of osteoporosis in postmenopausal women. The deficiency of estrogen leads to an increase in bone turnover. The apoptosis of osteoblasts increases, the time for bone formation shortens, and the apoptosis of osteoclasts decreases, thereby resulting in a decrease in bone density (7).

Menopausal women will experience lipid metabolism disorders due to the decrease in estrogen secretion (8). Recently, a single-center cross-sectional study analyzed the correlation between blood pressure and body composition in postmenopausal women with osteoporosis and hypertension, and found that body fat mass and visceral fat area were independent influencing factors for hypertension in postmenopausal osteoporosis patients (9). It remains unknown whether lipids are related to the blood pressure of postmenopausal women with osteoporosis. Therefore, we conducted this research.

## Methods and materials

### Study population

This is a secondary analysis of a retrospective cross-sectional study. The parent cohort initially enrolled 139 elderly hypertensive patients newly diagnosed with osteoporosis in the Department of Geriatrics Zhongshan Hospital Affiliated to Xiamen University between October 2022 and August 2023. Complete information about the study population has been previously described in detail (10). The original purpose of the parent cohort was to explore the correlation between vitamin D levels and blood pressure in elderly hypertensive patients with osteoporosis. We excluded 21 male patients with osteoporosis, aiming to conduct a survey on blood pressure status in postmenopausal osteoporosis patients.

Inclusion Criteria and Definitions: (1) Diagnosis of primary hypertension according to criteria outlined in the Chinese Guidelines for the Prevention and Treatment of Hypertension. This includes exclusion of hypertension caused by secondary factors, with systolic blood pressure (SBP) ≥ 140 mmHg and/or diastolic blood pressure (DBP) ≥ 90 mmHg recorded on three or more separate occasions, or use of antihypertensive medications; (2) Diagnosis of primary osteoporosis based on definitions provided in the Guidelines for the Diagnosis and Treatment of Primary Osteoporosis, characterized by systemic bone disease with low bone mass, increased bone fragility, and exclusion of secondary causes. Postmenopausal status was confirmed based on the following criteria: natural menopause for ≥1 year, with no menstrual bleeding in the past 12 months. This study primarily focused on postmenopausal osteoporosis.

Exclusion Criteria: (1) End-stage diseases, including heart failure (New York Heart Association [NYHA] class III–IV), severe renal insufficiency (CKD stages IV–V), severe hepatic dysfunction (Child-Pugh class C); (3) Malignant tumors, including hematological malignancies; (4) Acute phases of diseases such as acute organ failure, stroke, or trauma.

The previous study was approved by the Ethics Committee of Zhongshan Hospital Xiamen University. As no new participants were enrolled, according to local policies, re-application for ethical approval was not required, and the investigation complied with the Helsinki Declaration.

### Measurement of indicators

Blood pressure was measured using a non-invasive, portable, fully automated monitoring device (DMS-ABP, DMS Company, Beijing). A cuff of appropriate size was selected based on the patient’s arm circumference and worn on the left arm. Blood pressure monitoring was conducted from 8:00 a.m. to 8:00 a.m. the following day, with measurements taken every 30 min during the day and night. Valid readings were defined as achieving 70% or more of expected readings, with at least 20 valid readings during the day (8:00–22:00) and 7 at night (22:00–8:00 the next day). Cholesterol levels (TC, LDL-C, HDL-C) were measured from a single fasting venous blood sample drawn from each participant on the second morning after admission. The analysis was performed using a fully automated biochemical analyzer (Beckman Coulter AU5800, Germany) at the clinical testing center.

### Statistical analysis

The random forest imputation method was used to handle a small amount of missing data. The normality of the data distribution was assessed using the Shapiro–Wilk test. Data conforming to a normal distribution are presented as mean ± SD, while skewed distribution data sets are described as median [25th-75th percentile]. Shepherd’s Pi correlation analysis was used to explore the correlation between bone metabolic markers and lipid levels. A generalized additive model with linear regression was applied to examine the relationship between cholesterol and blood pressure, along with threshold effect analysis. The determination of breakpoints was performed by first identifying the positions where the slope of the curve exhibited the most significant changes. Subsequently, an iterative algorithm provided by the segmented package in R was applied to confirm the positions of these inflection points. Finally, adjustments were made to the breakpoints in consideration of clinical practicality, to ensure they are more applicable in real-world clinical settings. Statistical significance was set at *p* < 0.05 (two - sided). All statistical analyses were performed using R for Mac, version 4.2.2 (R Foundation for Statistical Computing, Vienna).

## Result

### Population characteristics

This retrospective study involved 118 postmenopausal women with osteoporosis and hypertension. They had a mean age of 73 and a relatively low BMI of 20.51 kg/m^2^ on average. Over half (53.4%) had peripheral arteriosclerosis, and nearly half (49.2%) had diabetes. Ambulatory blood pressure monitoring revealed most had a non - dipper blood pressure pattern, with elevated systolic blood pressure being prominent (24 h-mSBP: 139 ± 18 mmHg; diurnal mSBP: 140 ± 18 mmHg) and high nocturnal mSBP (136 ± 19 mmHg). Bone density was low in the lumbar spine and both femoral necks, and 25 - (OH) D3 levels were also low. The lipid profile of the overall population was as follows: the median TG was 1.36 mmol/L (IQR 0.88–1.73 mmol/L), and the mean TC, HDL-C, and LDL-C were 4.71 ± 1.26, 1.37 ± 0.34, and 3.03 ± 0.94 mmol/L, respectively. Table 1 shows the baseline characteristics of these patients.

**Table 1:** Patient demographics and baseline characteristics.

Characteristic	*N* = 118
Age (year)	73 ± 10
BMI, kg/m^2^	20.51 (19.25, 21.95)
Peripheral atherosclerosis	
No	55 (46.6%)
Yes	63 (53.4%)
CHD	
No	108 (91.5%)
Yes	10 (8.5%)
DM	
No	60 (50.8%)
Yes	58 (49.2%)
CKD	
No	111 (94.1%)
Yes	7 (5.9%)
Blood pressure pattern	
Dipper pattern	13 (11.0%)
Nondipper pattern	66 (55.9%)
Reverse dipper blood pressure	39 (33.1%)
24 h-mSBP, mmHg	139 ± 18
24 h- mDBP, mmHg	77 (70, 83)
Diurnal mSBP, mmHg	140 ± 18
Diurnal mDBP, mmHg	78 (70, 84)
Nocturnal mSBP, mmHg	136 ± 19
Nocturnal mDBP, mmHg	74 (66, 83)
NSBPDR, %	3 ± 8
NDBPDR, %	5 ± 8
Lumbar vertebra (T)	−2.50 (−3.40, −1.80)
Lumbar vertebra BMD	0.88 (0.79, 0.97)
Left neck of femur (T)	−2.60 (−3.00, −2.30)
Left neck of femur BMD	0.68 (0.62, 0.72)
Right neck of femur (T)	−2.60 (−3.00, −2.30)
Right neck of femur BMD	0.67 (0.62, 0.72)
Left hip (T)	−2.07 (−2.60, −1.61)
Left hip BMD	0.75 (0.67, 0.80)
Right hip (T)	−2.10 (−2.50, −1.60)
Right hip BMD	0.75 (0.70, 0.81)
25-(OH) D3, ng/mL	63 ± 21
IPTH, pg./mL	55 (39, 71)
β-Crosslaps, ng/mL	0.46 (0.24, 0.70)
Osteocalcin, ng/mL	16 (12, 21)
PINP, ng/mL	48 (34, 59)
WBC,109/L	6.11 (5.39, 7.21)
RBC,109/L	4.18 ± 0.53
Hemoglobin, g/L	127 (116, 134)
Platelet,1,012/L	217 ± 56
CRP, mg/L	2.8 (1.2, 4.2)
Albumin, g/L	39.6 ± 3.6
Total bilirubin, umol/L	10.9 (9.4, 13.5)
ALT, U/L	18 (13, 25)
AST, U/L	22 (18, 28)
TG, mmol/L	1.36 (0.88, 1.73)
TC, mmol/L	4.71 ± 1.26
HDL-C, mmol/L	1.37 ± 0.34
LDL-C, mmol/L	3.03 ± 0.94
Creatinine, umol/L	59 (51, 71)
BUN, mmol/L	5.73 (4.80, 7.20)
K+, mmol/L	3.94 (3.75, 4.15)
Na+, mmol/L	141.90 (140.30, 143.30)
D-Dimer, mg/L	0.44 (0.28, 0.82)

Mean ± SD; Median (25th-75th percentile); *n* (%).

CHD, coronary heart disease; DM, diabetes mellitus; CKD, chronic kidney disease; mSBP, mean systolic blood pressure; mDBP, mean diastolic blood pressure; NSBPDR, nocturnal systolic blood pressure drop rate; NDBPDR: nocturnal diastolic blood pressure drop rate; BMD, bone mineraldensity; WBC, White blood cell; RBC, Red blood cell; IPTH, intact parathyroid hormone; PINP, Procollagen I N-Terminal Propeptide; ALT, alanine aminotransferase; AST, aspartate aminotransferase; TG, triglyceride; TC, total cholesterol; LDL-C: low density lipoprotein cholesterol; HDL-C, high density lipoprotein cholesterol; BUN: blood urea nitrogen; CRP, C-reactive protein.

### Shepherd’s Pi correlation analysis

**Figure 1:**
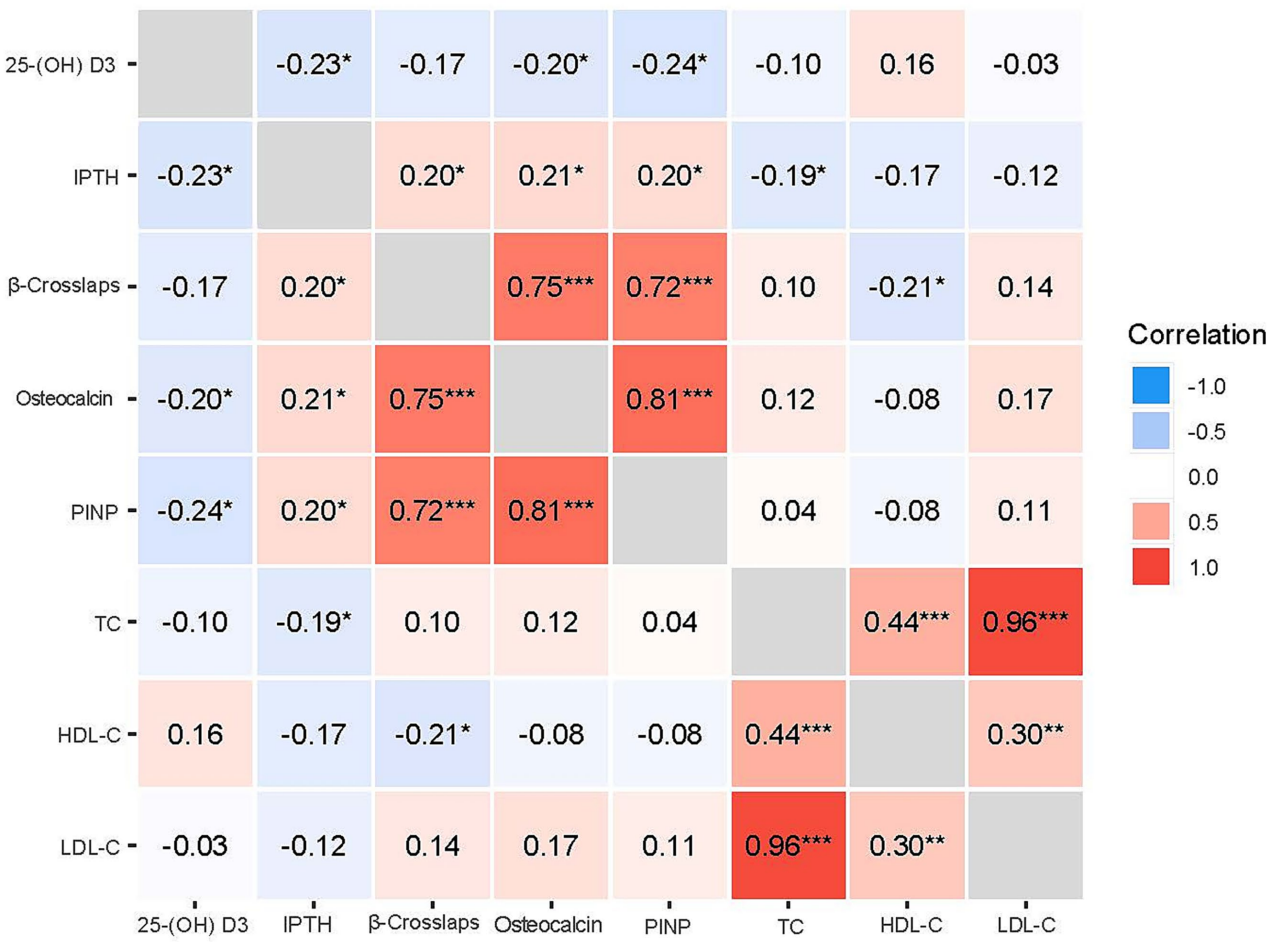
Shepherd’s Pi correlation matrix between bone metabolic markers and lipid parameters. This figure visualizes the correlation coefficients between bone metabolic markers and lipid parameters. Blue shades indicate positive correlations, while red shades indicate negative correlations. The intensity of the color corresponds to the strength of the correlation. TC, total cholesterol; LDL-C, low-density lipoprotein cholesterol; HDL-C, high-density lipoprotein cholesterol; TG, triglycerides; IPTH, intact parathyroid hormone; PINP: Procollagen I N-Terminal Propeptide.

Shepherd’s Pi correlation analysis was used to explore the correlation between cholesterol and bone metabolic indicators (11). Shepherd’s Pi correlation analysis was conducted with the easystats package in R. This method is equivalent to a Spearman’s rank correlation after outlier removal by means of bootstrapped Mahalanobis distance, providing a robust correlation measure through identification and removal of multivariate outliers. Results showed that TC had a negative correlation with IPTH (*r* = −0.19), and HDL-C also had a certain negative correlation with β - crosslaps (*r* = −0.21). The specific results are shown in Figure 1 and Supplementary Table S2.

### Relationship between TC and blood pressure

Multicollinearity among the variables incorporated into the GAM was assessed using the variance inflation factor (VIF) and tolerance. These same variables were then carried forward for further threshold effect analysis. The judgment criteria were as follows: a VIF < 5 was considered to indicate low multicollinearity, a VIF between 5 and 10 suggested a certain degree of multicollinearity, and a VIF ≥ 10 implied severe multicollinearities. In the present study, among the covariates included in the GAM, only creatinine (VIF = 5.48) exhibited a certain degree of multicollinearity, while no significant multicollinearity was observed for the other indicators. For detailed results, please refer to Supplementary Table S1.

**Figure 2:**
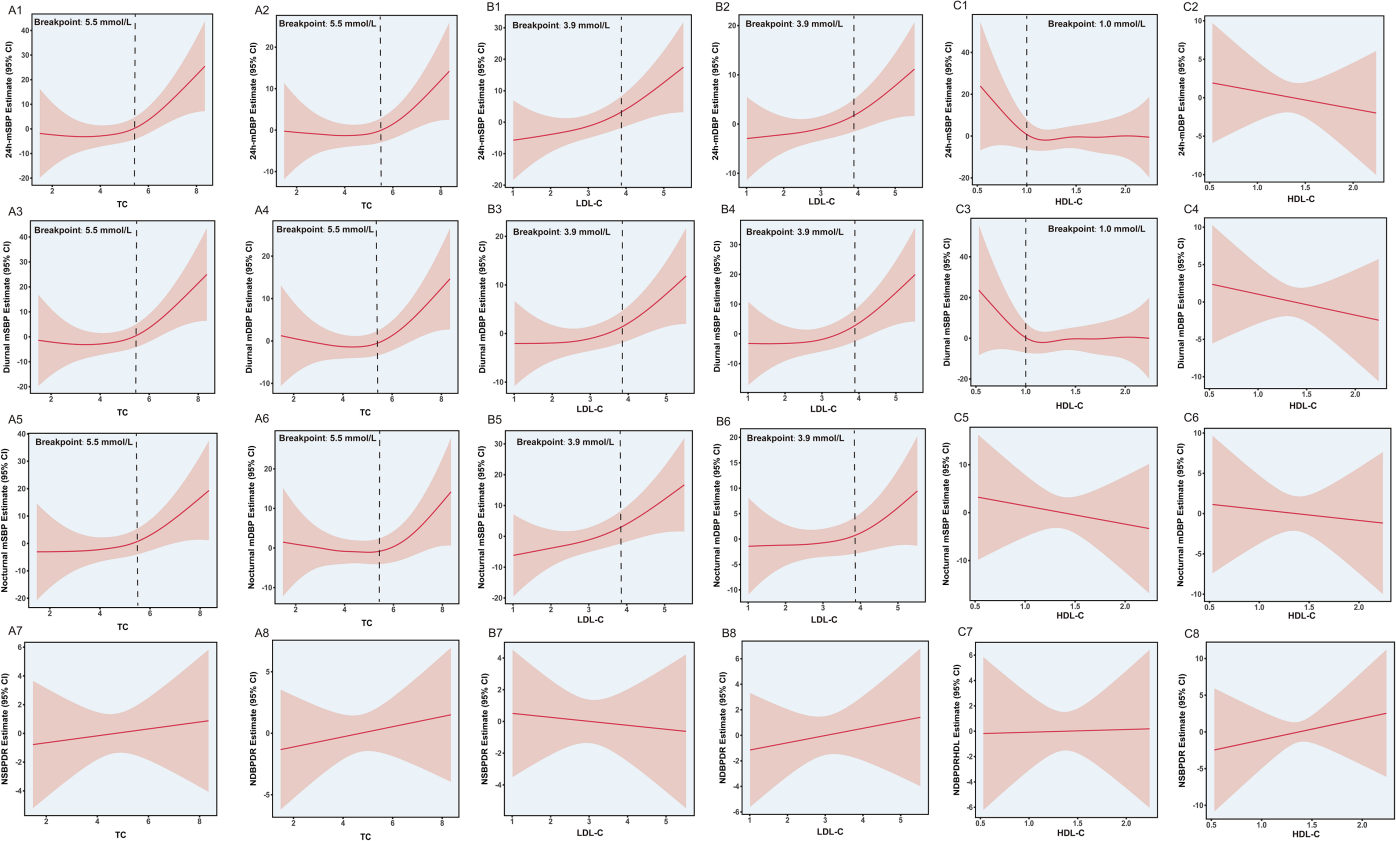
The results of the generalized additive model fitted with linear regression. TC, total cholesterol; LDL-C, low-density lipoprotein cholesterol; HDL-C, high-density lipoprotein cholesterol; NSBPDR: nocturnal systolic blood pressure drop rate; NDBPDR: nocturnal diastolic blood pressure drop rate.

The non-linear relationship was identified between TC and multiple blood pressure parameters using the GAM. This was observed for 24 h-mSBP, 24 h-mDBP, diurnal mean SBP/DBP, and nocturnal mean SBP/DBP, but not for the nocturnal systolic or diastolic blood pressure decline rates, as shown in Figures 2A1–A8. Further threshold effect analysis revealed significant differences between the standard linear regression and piecewise linear regression models. For 24 h-mSBP, the standard linear regression model showed a significant positive association (*β* = 3.45, 95% CI: 0.50–6.40; *p* = 0.024). The piecewise linear regression model, with a breakpoint at TC = 5.5 mmol/L, indicated no significant association when TC < 5.5 mmol/L (*β* = −0.71, 95% CI: −4.93-3.50; *p* = 0.739), but a significant positive association when TC ≥ 5.5 mmol/L (*β* = 12.62, 95% CI 5.23–20.00; *p* < 0.001). This means that 29 out of 118 participants (24.6%) had a TC ≥ 5.5 mmol/L, and for each 1 mmol/L increase in TC, the 24 h-mSBP increased by 12.62 mmHg. Similar patterns were observed for diurnal mSBP and nocturnal mSBP, where significant associations were found only when TC ≥ 5.5 mmol/L. The log-likelihood ratio tests indicated that the piecewise linear regression models provided a better fit for the data compared to the standard linear regression models. These findings suggest a potential threshold effect of TC on blood pressure, particularly for systolic blood pressure. The results are shown in Table 2.

**Table 2:** Threshold effect analysis of TC on blood pressure.

24 h-mSBP (mmHg)	Beta (95% CI)*	*p*-value
Fitting by standard linear regression model	3.45 (0.50, 6.40)	0.024
Fitting by piecewise linear regression model (break-point = 5.5)		
TC < 5.5 mmol/L	−0.71 (−4.93, 3.50)	0.739
TC ≥ 5.5 mmol/L	12.62 (5.23, 20.00)	<0.001
Log likelihood ratio		0.003
24 h-mDBP (mmHg)		
Fitting by standard linear regression model	1.60 (−0.28, 3.48)	0.099
Fitting by piecewise linear regression model (break-point = 5.5)		
TC < 5.5 mmol/L	−0.87(−3.57, 1.83)	0.529
TC ≥ 5.5 mmol/L	7.03 (2.30, 11.78)	0.004
Log likelihood ratio		0.007
Diurnal mSBP (mmHg)		
Fitting by standard linear regression model	3.39 (0.36, 6.41)	0.031
Fitting by piecewise linear regression model (break-point = 5.5)		
TC < 5.5 mmol/L	−0.80 (−5.12, 3.53)	0.719
TC ≥ 5.5 mmol/L	12.60 (5.00, 20.19)	0.001
Log likelihood ratio		0.004
Diurnal mDBP (mmHg)		
Fitting by standard linear regression model	1.04 (−1.01, 3.09)	0.324
Fitting by piecewise linear regression model (break-point = 5.5)		
TC < 5.5 mmol/L	−1.35 (−4.31, 1.62)	0.374
TC ≥ 5.5 mmol/L	6.28 (1.08, 11.48)	0.018
Log likelihood ratio		0.016
Nocturnal mDBP (mmHg)		
Fitting by standard linear regression model	1.04 (−1.01, 3.09)	0.324
Fitting by piecewise linear regression model (break-point = 5.5)		
TC < 5.5 mmol/L	−1.35 (−4.31, 1.62)	0.374
TC ≥ 5.5 mmol/L	6.28 (1.08, 11.48)	0.018
Log likelihood ratio		0.016
Nocturnal mSBP (mmHg)		
Fitting by standard linear regression model	2.80 (−0.35, 5.95)	0.085
Fitting by piecewise linear regression model (break-point = 5.5)		
TC < 5.5 mmol/L	−0.71 (−5.27, 3.85)	0.761
TC ≥ 5.5 mmol/L	10.51 (2.51, 18.51)	0.010
Log likelihood ratio		0.022
NDBPDR (%)		
Fitting by standard linear regression model	−2.25 (−12.16, 7.65)	0.657
NSBPDR (%)		
Fitting by standard linear regression model	0.24 (−1.06, 1.55)	0.718

*Adjusted for: Age, BMI, Peripheral atherosclerosis, CHD, DM, CKD, 25-(OH) D3, IPTH, β-crosslaps, Osteocalcin, PINP, CRP, Albumin, TG, creatinine, BUN, K+, Na+, D-Dimer, LDL, WBC, RBC, HGB, PLT.

CHD, coronary heart disease; DM, diabetes mellitus; CKD, chronic kidney disease; mSBP, mean systolic blood pressure; mDBP, mean diastolic blood pressure; NSBPDR, nocturnal systolic blood pressure drop rate; NDBPDR, nocturnal diastolic blood pressure drop rate; WBC, White blood cell; RBC, Red blood cell; IPTH, intact parathyroid hormone; PINP, Procollagen I N-Terminal Propeptide; TG, triglyceride; TC, total cholesterol; HDL-C, high density lipoprotein cholesterol; BUN, blood urea nitrogen; CRP, C-reactive protein; HBG, hemoglobin; PLT, platelet.

### Relationship between LDL-C and blood pressure

The exploration of the non-linear relationship between LDL-C and blood pressure parameters using a GAM revealed non-linear associations with all blood pressure parameters, with the exception of the NSBPDR and NDBPDR. The results are shown in Figures 2B1–B8. Similar to the threshold effect analysis of TC, the analysis of LDL-C also revealed significant differences between the standard linear regression and piecewise linear regression models. For 24 h-mSBP, the standard linear regression model showed a significant positive association with LDL-C (*β* = 4.82, 95% CI: 0.60–9.04; *p* = 0.028). In contrast, the piecewise linear regression model—with a breakpoint identified at LDL-C = 3.9 mmol/L—indicated no significant association when LDL-C < 3.9 mmol/L (*β* = 0.93, 95% CI: −4.39–6.26; *p* = 0.731). Notably, a total of 18 participants (15.2%) in the cohort had LDL-C ≥ 3.9 mmol/L, and for participants with LDL-C at this concentration or above, each 1 mmol/L increase in LDL-C was significantly associated with an 18.33 mmHg increase in 24 h-mSBP (β = 18.33, 95% CI: 5.93–30.74; *p* = 0.004). Similar patterns were observed for 24 h-mDBP, diurnal mSBP, diurnal mDBP, nocturnal mSBP, and nocturnal mDBP, with detailed beta coefficients and confidence intervals provided in Table 3. The log-likelihood ratio tests indicated that the piecewise linear regression models provided a better fit for the data compared to the standard linear regression models. These findings suggest a potential threshold effect of LDL-C on blood pressure, with significant associations observed only when LDL-C levels exceeded the breakpoint of 3.9 mmol/L.

**Table 3:** Threshold effect analysis of LDL-C on blood pressure.

24 h-mSBP (mmHg)	Beta (95% CI)*	*P*-value
Fitting by standard linear regression model	4.82 (0.60, 9.04)	0.028
Fitting by piecewise linear regression model (break-point = 3.9)		
LDL-C < 3.9 mmol/L	0.93 (−4.39, 6.26)	0.731
LDL-C ≥ 3.9 mmol/L	18.33 (5.93, 30.74)	0.004
Log likelihood ratio		0.011
24 h-mDBP (mmHg)		
Fitting by standard linear regression model	2.78 (0.10, 5.47)	0.045
Fitting by piecewise linear regression model (break-point = 3.9)		
LDL-C < 3.9 mmol/L	0.60 (−2.81, 4.01)	0.728
LDL-C ≥ 3.9 mmol/L	10.36 (2.42, 18.30)	0.011
Log likelihood ratio		0.025
Diurnal mSBP (mmHg)		
Fitting by standard linear regression model	4.62 (0.28, 8.95)	0.040
Fitting by piecewise linear regression model (break-point = 3.9)		
LDL-C < 3.9 mmol/L	0.77 (−4.70, 6.25)	0.782
LDL-C ≥ 3.9 mmol/L	17.97 (5.21, 30.73)	0.006
Log likelihood ratio		0.014
Diurnal mDBP (mmHg)		
Fitting by standard linear regression model	2.74 (0.01, 5.46)	0.052
Fitting by piecewise linear regression model (break-point = 3.9)		
LDL-C < 3.9 mmol/L	0.39 (−3.06, 3.84)	0.826
LDL-C ≥ 3.9 mmol/L	10.91 (2.88, 18.95)	0.008
Log likelihood ratio		0.017
Nocturnal mSBP (mmHg)		
Fitting by standard linear regression model	4.66 (0.17, 9.16)	0.045
Fitting by piecewise linear regression model (break-point = 3.9)		
LDL-C < 3.9 mmol/L	1.44 (−4.30, 7.18)	0.623
LDL-C ≥ 3.9 mmol/L	15.87 (2.51 29.23)	0.020
Log likelihood ratio		0.049
Nocturnal mDBP (mmHg)		
Fitting by standard linear regression model	1.83 (−1.11, 4.77)	0.226
Fitting by piecewise linear regression model (break-point = 3.9)		
LDL-C < 3.9 mmol/L	−0.38 (−4.13, 3.36)	0.841
LDL-C ≥ 3.9 mmol/L	9.51 (0.79, 18.23)	0.032
Log likelihood ratio		0.039
NSBPDR (%)		
Fitting by standard linear regression model	−0.25 (−2.12, 1.62)	0.793
NDBPDR (%)		
Fitting by standard linear regression model	0.59 (−1.51, 2.64)	0.594

*Adjusted for: Age, BMI, Peripheral atherosclerosis, CHD, DM, CKD, 25-(OH) D3, IPTH, β-crosslaps, Osteocalcin, PINP, CRP, Albumin, TG, creatinine, BUN, K+, Na+, D-Dimer, LDL, WBC, RBC, HGB, PLT.

CHD, coronary heart disease; DM, diabetes mellitus; CKD, chronic kidney disease; mSBP, mean systolic blood pressure; mDBP, mean diastolic blood pressure; NSBPDR, nocturnal systolic blood pressure drop rate; NDBPDR, nocturnal diastolic blood pressure drop rate; WBC, White blood cell; RBC, Red blood cell; IPTH, intact parathyroid hormone; PINP, Procollagen I N-Terminal Propeptide; TG, triglyceride; TC, total cholesterol; HDL-C, high density lipoprotein cholesterol; BUN, blood urea nitrogen; CRP, C-reactive protein; HBG, hemoglobin; PLT, platelet; HDL-C, high density lipoprotein cholesterol.

### Relationship between HDL-C and blood pressure

The generalized additive model was used to explore the nonlinear relationship between HDL-C and blood pressure indicators, and the results showed that only 24 h-mSBP and diurnal mSBP had a nonlinear relationship. Further threshold analysis shows that: for 24 h-mSBP, the standard linear regression model showed no significant association with HDL-C (*β* = −0.14, 95% CI: −4.88 to 4.61; *p* = 0.955). In contrast, the piecewise linear regression model—with an optimal breakpoint identified at HDL-C = 1 mmol/L—revealed distinct associations based on HDL-C levels relative to this threshold. Notably, 11 participants (9.3%) in the cohort had HDL-C < 1 mmol/L; for this range, each 0.34 mmol/L increase in HDL-C (equivalent to the standard deviation of HDL-C in the overall cohort) was significantly associated with a 32.13 mmHg decrease in 24 h-mSBP (*β* = −32.13, 95% CI: −57.67 to −6.60; *p* = 0.014). When HDL-C ≥ 1 mmol/L, however, no significant association with 24 h-mSBP was observed (*β* = 0.69, 95% CI: −3.97 to 5.35; *p* = 0.773). Similar patterns of association between HDL-C and systolic blood pressure were observed for diurnal mSBP. The detailed results are presented in Table 4 and Figures 2C1–C8.

**Table 4:** Threshold effect analysis of HDL-C on blood pressure.

24 h-mSBP (mmHg)	Beta (95% CI)*	*P*-value
Fitting by standard linear regression model	−0.14 (−4.88, 4.61)	0.955
Fitting by piecewise linear regression model (break-point = 1)		
HDL-C < 1	−32.13 (−57.67, −6.60)	0.014
HDL-C ≥ 1	0.69 (−3.97, 5.35)	0.773
Log likelihood ratio		0.005
24 h-mDBP (mmHg)		
Fitting by standard linear regression model	−0.77 (−3.79, 2.25)	0.619
Diurnal mSBP (mmHg)		
Fitting by standard linear regression model	0.14 (−4.73, 5.01)	0.955
Fitting by piecewise linear regression model (break-point = 1)		
HDL-C < 1	−32.43 (−58.66, −6.21)	0.015
HDL-C ≥ 1	0.98 (−3.81, 5.77)	0.688
Log likelihood ratio		0.006
Diurnal mDBP (mmHg)		
Fitting by standard linear regression model	−0.94 (−4.01, 2.12)	0.548
Nocturnal mDBP (mmHg)		
Fitting by standard linear regression model	−0.45 (−3.76, 2.85)	0.789
Nocturnal mSBP (mmHg)		
Fitting by standard linear regression model	−1.29 (−6.34, 3.77)	0.619
NSBPDR (%)		
Fitting by standard linear regression model	1.06 (−1.04, 3.16)	0.327
NDBPDR (%)		
Fitting by standard linear regression model	0.07 (−2.26, 2.41)	0.951

*Adjusted for: Age, BMI, Peripheral atherosclerosis, CHD, DM, CKD, 25-(OH) D3, IPTH, β-crosslaps, Osteocalcin, PINP, CRP, Albumin, TG, creatinine, BUN, K+, Na+, D-Dimer, LDL, WBC, RBC, HGB, PLT.

CHD, coronary heart disease; DM, diabetes mellitus; CKD, chronic kidney disease; mSBP, mean systolic blood pressure; mDBP, mean diastolic blood pressure; NSBPDR, nocturnal systolic blood pressure drop rate; NDBPDR, nocturnal diastolic blood pressure drop rate; WBC, White blood cell; RBC, Red blood cell; IPTH, intact parathyroid hormone; PINP, Procollagen I N-Terminal Propeptide; TG, triglyceride; LDL-C, low density lipoprotein cholesterol; BUN, blood urea nitrogen; CRP, C-reactive protein; HBG, hemoglobin; PLT, platelet.

## Discussion

Blood lipids play a crucial role in the occurrence and progression of many diseases. Studies have found that LDL-C and HDL-C may be associated with the occurrence of osteoporosis in postmenopausal women (12). Furthermore, it has been discovered that cholesterol is related to the control of blood pressure (13). In this retrospective study, we included 118 postmenopausal female patients with hypertension and osteoporosis. We applied both standard linear regression and piecewise linear regression to analyze the correlation between blood pressure and blood lipids. The results showed that the piecewise linear regression model provided a better fit to the data. After determining the optimal cutoff value, we found that when TC ≥ 5.5 mmol/L or LDL-C ≥ 3.9 mmol/L, blood pressure showed a positive correlation with blood lipids, including 24 h-mSBP, 24 h-mDBP, diurnal mSBP, diurnal mDBP, nocturnal mDBP and nocturnal mSBP. When HDL-C is<1 mmol/L, systolic blood pressure is negatively correlated with HDL-C, while no correlation was found for diastolic blood pressure. When TC<5.5 mmol/L, LDL-C<3.9 mmol/L, or HDL-C ≥ 1 mmol/L, no correlation was found between blood pressure and lipid levels. These findings provide important reference values for the management of blood lipids and blood pressure in postmenopausal osteoporotic patients. In the future, clinicians should fully consider this threshold effect between lipid levels and blood pressure when assessing cardiovascular risk in these patients, and develop more precise and individualized intervention strategies to improve long-term prognosis.

Postmenopausal women are more prone to osteoporosis and cardiovascular diseases due to the decrease in estrogen levels (14, 15). Estrogen is produced in the ovaries using LDL-C as a substrate. However, during menopause, LDL-C cannot be utilized to generate estrogen. Therefore, the level of LDL-C in postmenopausal women usually increases (16). Meng et al. discovered through animal experiments that the levels of TC and LDL-C in ovariectomized mice increased, which might be related to the decline in the function of estradiol and its receptors (17–19). Therefore, postmenopausal women are more prone to developing dyslipidemia (20).

Dyslipidemia is associated with osteoporosis. ALAY et al. revealed that in postmenopausal women, LDL-C was negatively correlated with the bone density of L2-L4 (21). In another experiment, Sage et al. found that dyslipidemia could inhibit the proliferation of osteoblasts in mice (22). The impact of blood lipids on bone density may occur through the accumulation of oxidized lipids in micro vessels, which promotes the release of inflammatory factors, thereby causing changes in the bone microenvironment and ultimately leading to osteogenesis disorders (12). Our study found a modest inverse association between TC and IPTH, and a negative correlation between HDL-C and the bone resorption marker β-crosslaps. These specific correlations indicate a potential link between lipid profiles and bone metabolism, aligning with previous reports that hypercholesterolemia can influence bone turnover (23).

Dyslipidemia is a significant risk factor for cardiovascular diseases, especially coronary atherosclerotic heart disease (24). The INTERHEART study found that patients with both dyslipidemia and hypertension have a significantly increased risk of cardiovascular diseases (25). Furthermore, studies have shown that treating both hypertension and dyslipidemia simultaneously can significantly reduce the risk of cardiovascular diseases (26, 27). The underlying mechanism may be related to endothelial dysfunction. The vascular endothelial dysfunction caused by hypertension will accelerate the harmful consequences resulting from dyslipidemia (28). And the vascular remodeling caused by abnormal blood lipids further aggravated hypertension (29). Similarly, our research results also indicate that there are correlations between lipid indicators and blood pressure.

Postmenopausal women are more susceptible to dyslipidemia, a condition that can contribute to the development of osteoporosis and may also lead to elevated blood pressure. Furthermore, dyslipidemia and hypertension can act synergistically, significantly increasing the overall risk of cardiovascular diseases. Our research has revealed that there is a correlation between the blood lipid levels and blood pressure in female patients with hypertension who also have osteoporosis. Further Prospective research is needed in the future to confirm whether the strategy of simultaneously reducing both cholesterol and blood pressure can effectively lower the cardiovascular disease risk for such patients.

### Limitation

We acknowledge several inherent limitations in this study. First, as a secondary analysis of a retrospective, cross-sectional study, we are unable to establish a causal relationship between cholesterol levels and ambulatory blood pressure; our findings only suggest a potential association. Second, as this is a secondary analysis of a parent cohort focused on vitamin D, some key clinical variables (e.g., detailed medication history, estrogen use) were not systematically collected, which may introduce the risk of selective bias. Finally, our study population was limited to postmenopausal women with osteoporosis. Finally, our study population was limited to postmenopausal women with osteoporosis; therefore, the generalizability of our findings to other populations remains to be determined.

## Conclusion

This study explored the relationship between cholesterol levels and ambulatory blood pressure in postmenopausal osteoporotic patients. The results indicated that TC ≥ 5.5 mmol/L and LDL-C ≥ 3.9 mmol/L were positively associated with blood pressure, whereas HDL-C < 1 mmol/L was negatively correlated with systolic blood pressure. These findings suggest a potential threshold effect between lipid levels and blood pressure. The study provides valuable insights for clinicians in managing cardiovascular risk in postmenopausal osteoporotic patients. Further research is needed to confirm the causal relationship and evaluate the effectiveness of lipid-lowering interventions on blood pressure control.

## Data Availability

The raw data supporting the conclusions of this article will be made available by the authors, without undue reservation.
