# Supplementary material for: Correlation between cholesterol and ambulatory blood pressure in postmenopausal osteoporotic patients: a post-hoc analysis of an observational study
Source: Front Med (Lausanne). 2025 Dec 8;12:1662973. doi: 10.3389/fmed.2025.1662973 (PMC12719291; doi:10.3389/fmed.2025.1662973)
Supplement: Supplementary file 1 [file Image_1.PDF]

Validation Results and Model Information of the Generalized Additive Model

Goodness-of-fit Assessment of GAM Models between TC and Blood Pressure Parameters

Model	R^2	Deviance explained	GCV	AIC	BIC	Log-likelihood	n
24h-mSBP	0.1371	0.3224	358.7568	1,024.21	1,099.37	-484.98	118
24h-mDBP	0.1384	0.3235	146.9761	918.90	994.09	-432.32	118
Diurnal mSBP	0.1355	0.3209	378.4466	1,030.54	1,105.59	-488.18	118
Diurnal mDBP	0.1071	0.2992	150.1471	921.40	996.68	-433.53	118
Nocturnal mSBP	0.0524	0.2538	417.1951	1,042.17	1,116.59	-494.22	118
Nocturnal mDBP	0.0040	0.2200	175.1896	939.49	1,015.33	-442.37	118
NSBPDR	0.0220	0.2226	72.5999	836.31	908.34	-392.15	118
NDBPDR	-0.0452	0.1692	89.0655	860.43	932.47	-404.21	118

GCV: Generalized Cross-Validation; AIC: Akaike Information Criterion; BIC: Bayesian Information Criterion; NSBPDR: Nocturnal systolic blood pressure decline rate ;NDBPDR: Nocturnal diastolic blood pressure decline rate

1) GAM model validation focusing on 24h mSBP and TC

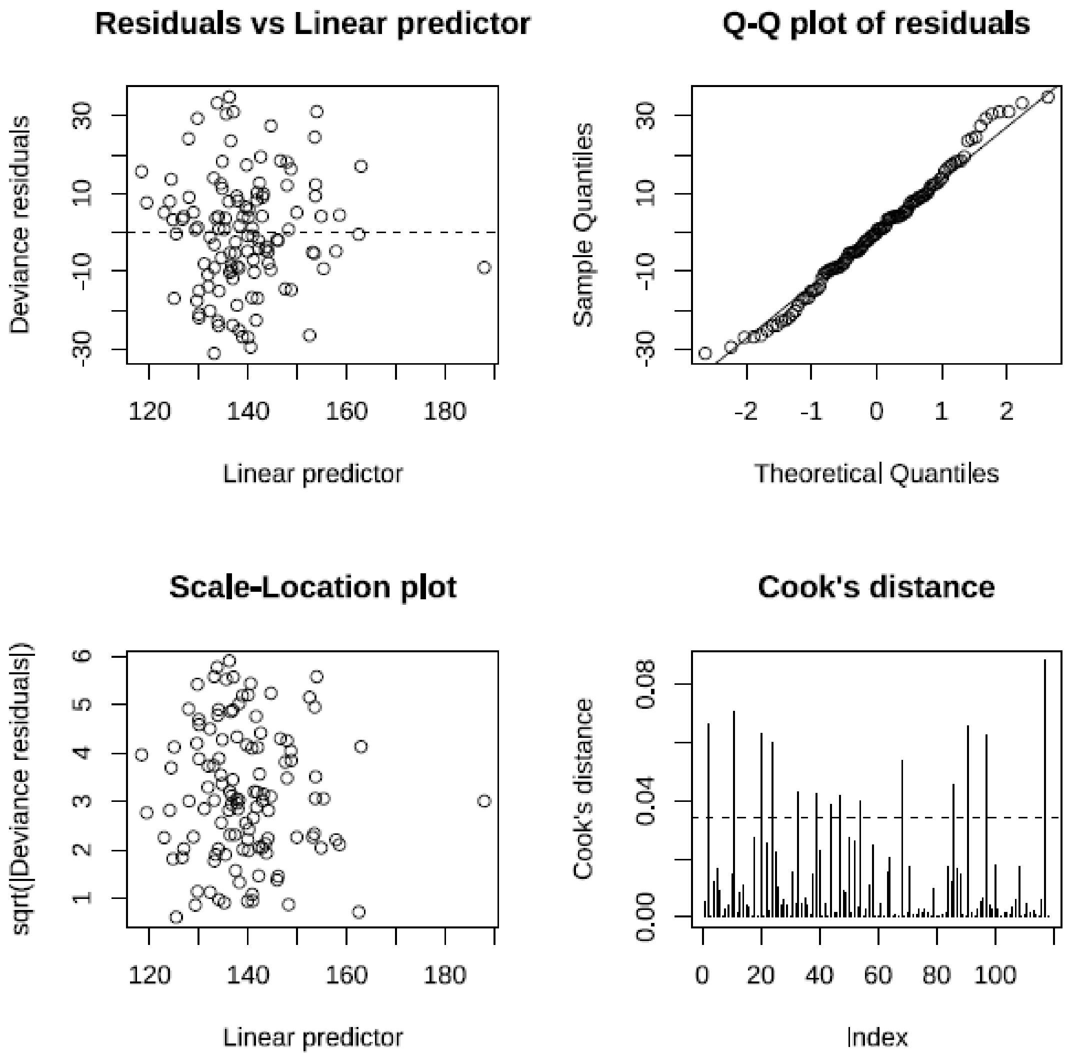

2) GAM model validation focusing on 24h mDBP and TC

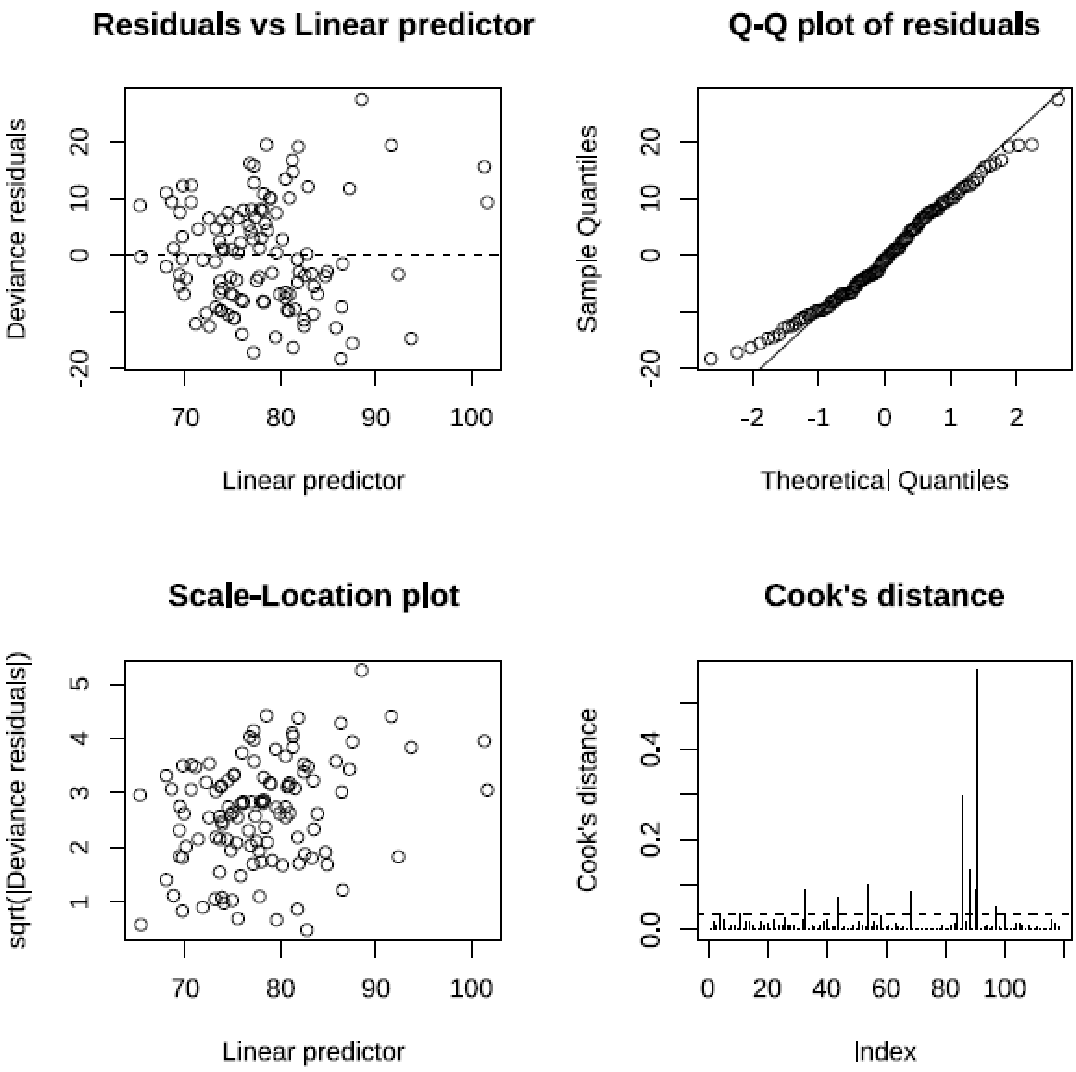

3) GAM model validation focusing on Diurnal mSBP and TC

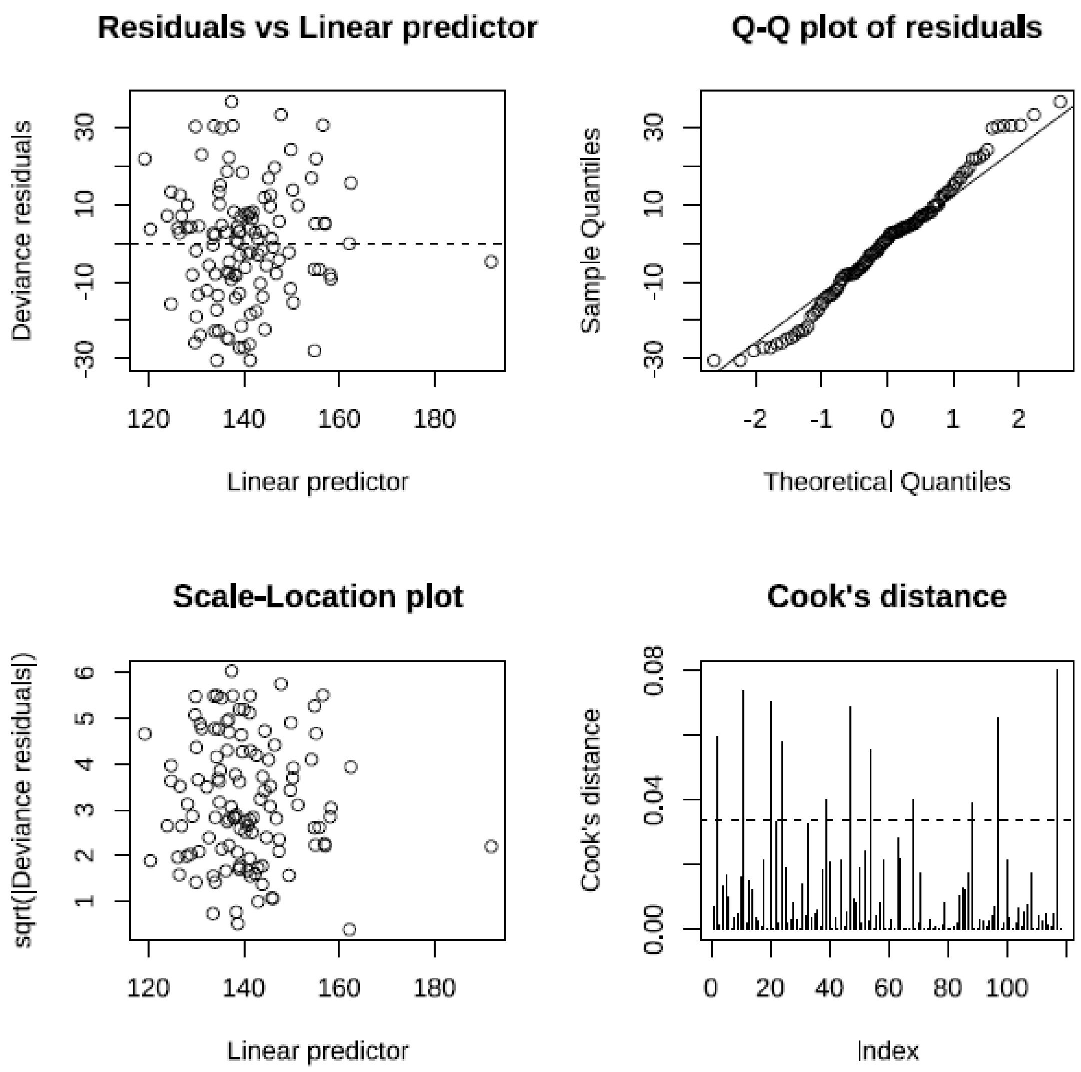

4) GAM model validation focusing on Diurnal mDBP and TC

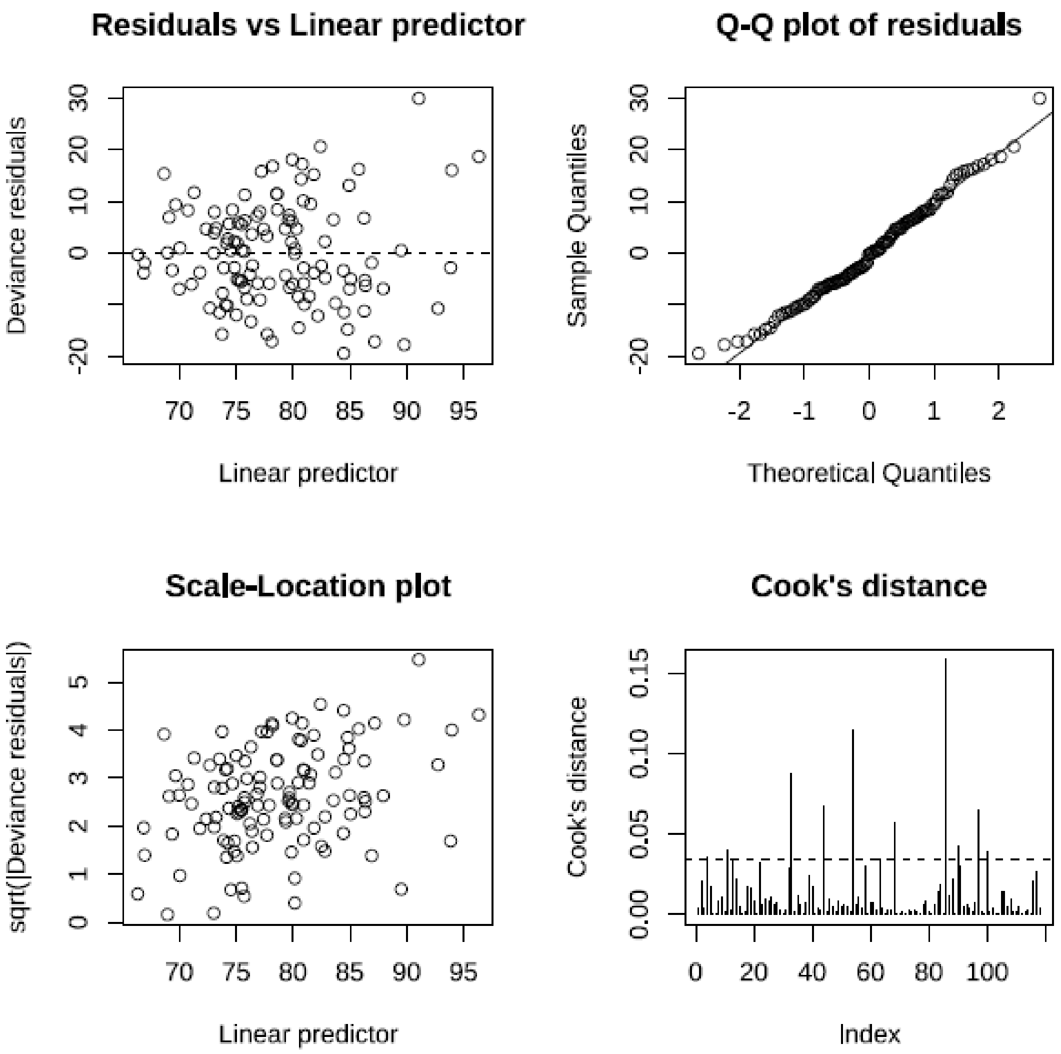

5) GAM model validation focusing on Nocturnal mDBP and TC

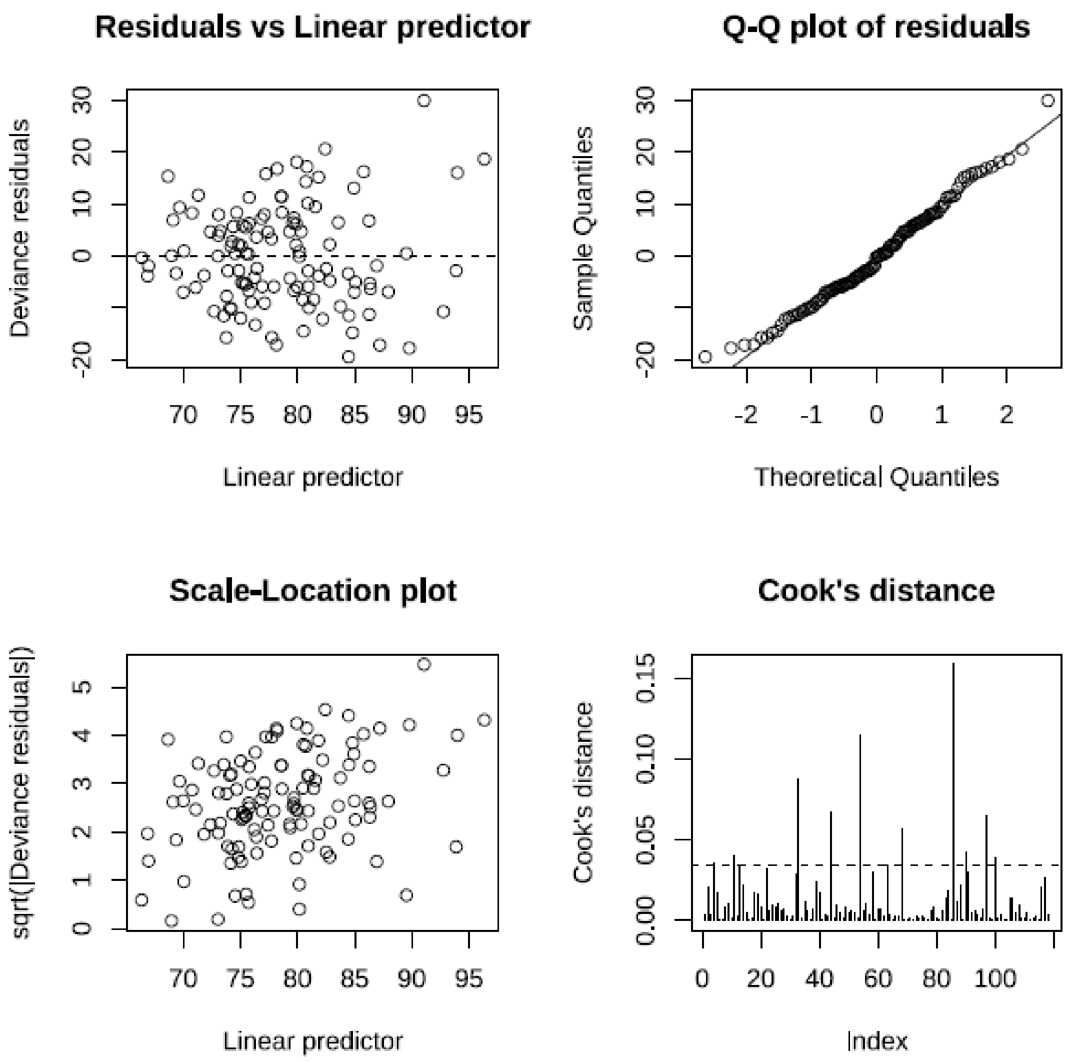

6) GAM model validation focusing on Nocturnal mSBP and TC

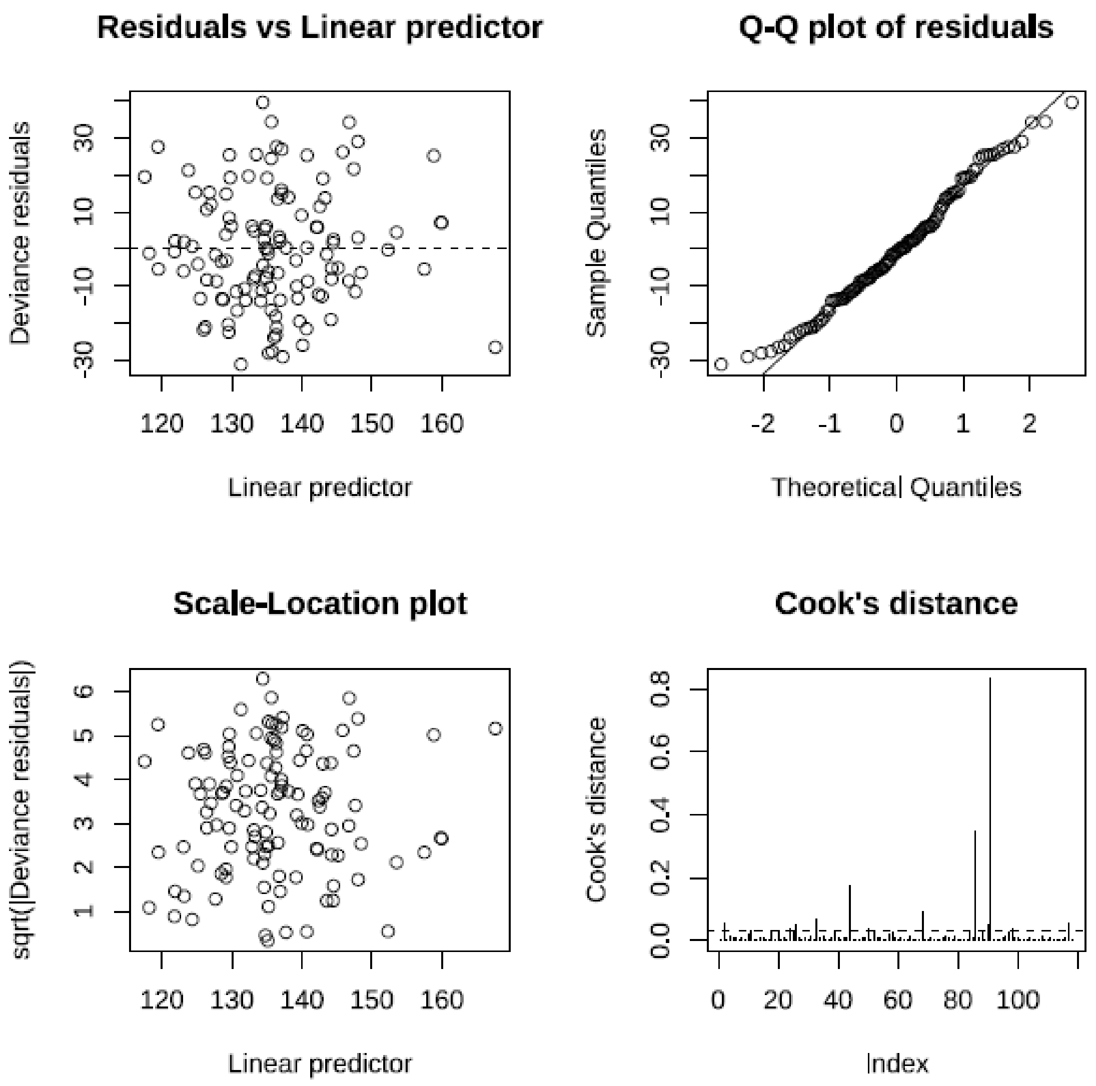

7) GAM model validation focusing on NSBPDR and TC

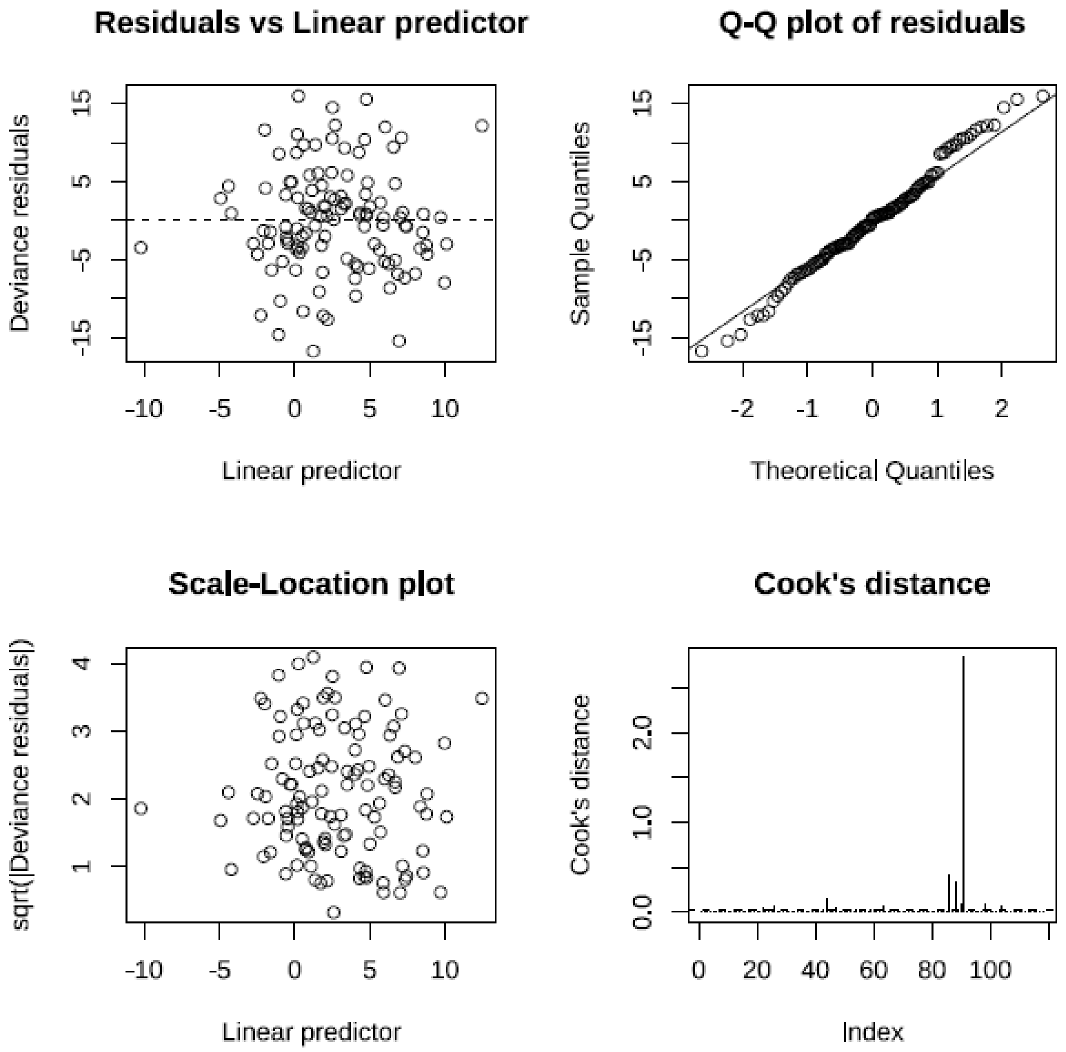

8) GAM model validation focusing on NDBPDR and TC

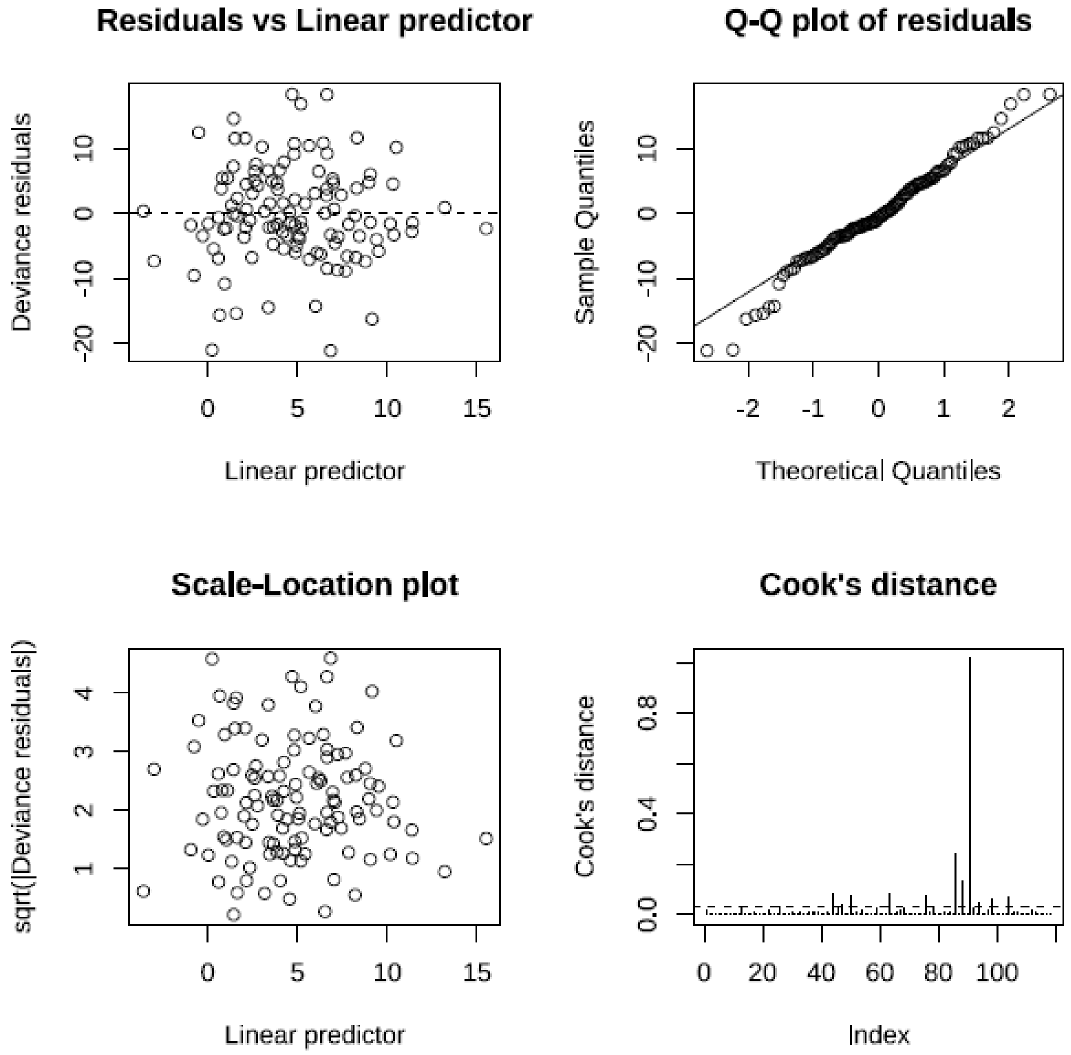

Goodness-of-fit Assessment of GAM Models between LDL-C and Blood Pressure Parameters

Model	R2	Deviance explained	GCV	AIC	BIC	Log-likelihood	n
24h-mSBP	0.1261	0.3200	366.6346	1,026.29	1,103.75	-485.19	118
24h-mDBP	0.1260	0.3188	150.1759	921.06	998.10	-432.72	118
Diurnal mSBP	0.1233	0.3174	387.2457	1,032.77	1,110.10	-488.48	118
Diurnal mDBP	0.0927	0.2934	153.7666	923.80	1,001.05	-434.02	118
Nocturnal mSBP	0.0469	0.2557	423.1839	1,043.41	1,119.97	-494.07	118
Nocturnal mDBP	-0.0223	0.2035	180.7592	942.91	1,020.06	-443.61	118
NSBPDR	0.0206	0.2299	73.4887	837.19	912.00	-391.60	118
NDBPDR	-0.0556	0.1700	90.9250	862.31	937.12	-404.16	118

GCV: Generalized Cross-Validation; AIC: Akaike Information Criterion; BIC: Bayesian Information Criterion; NSBPDR: Nocturnal systolic blood pressure decline rate; NDBPDR: Nocturnal diastolic blood pressure decline rate

9) GAM model validation focusing on 24h mSBP and LDL-C

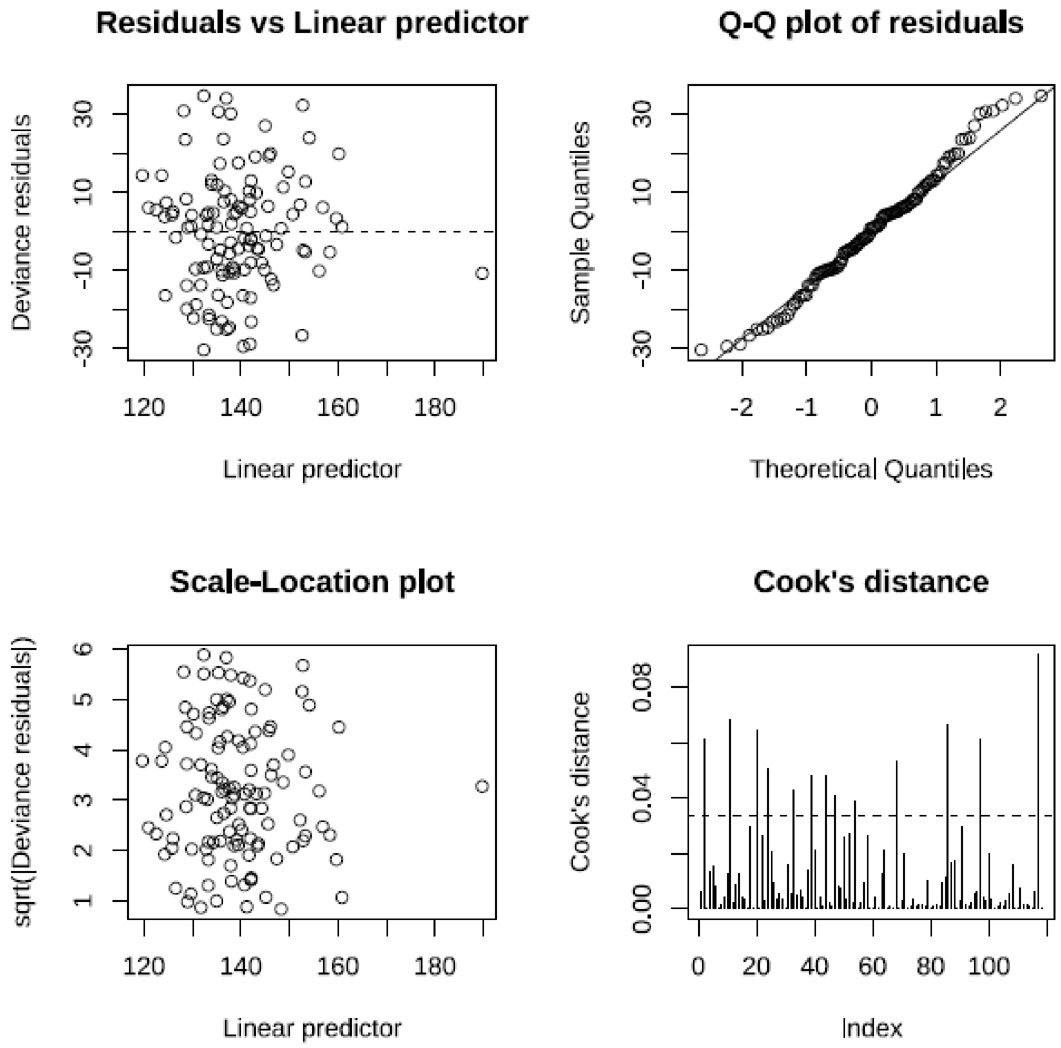

10) GAM model validation focusing on 24h mDBP and LDL-C

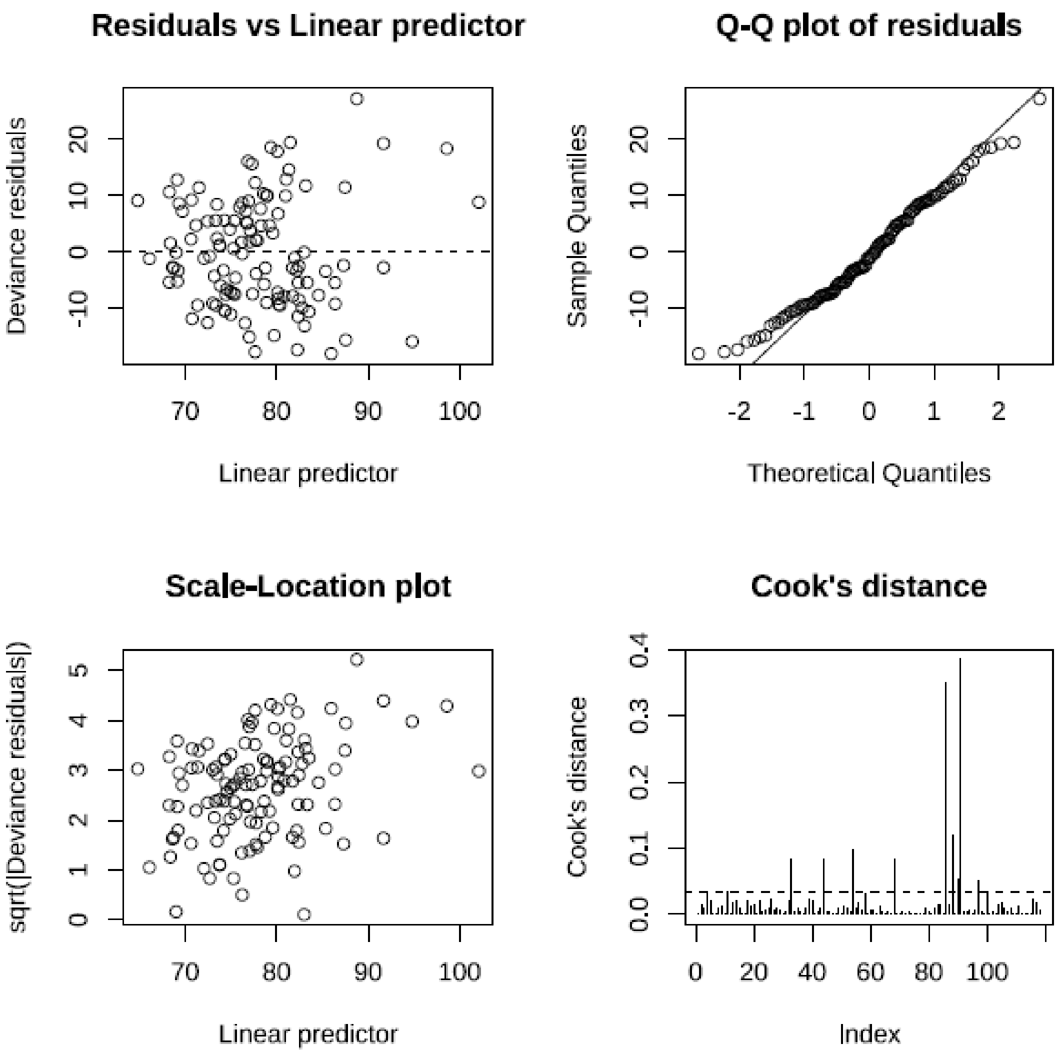

11) GAM model validation focusing on Diurnal mSBP and LDL-C

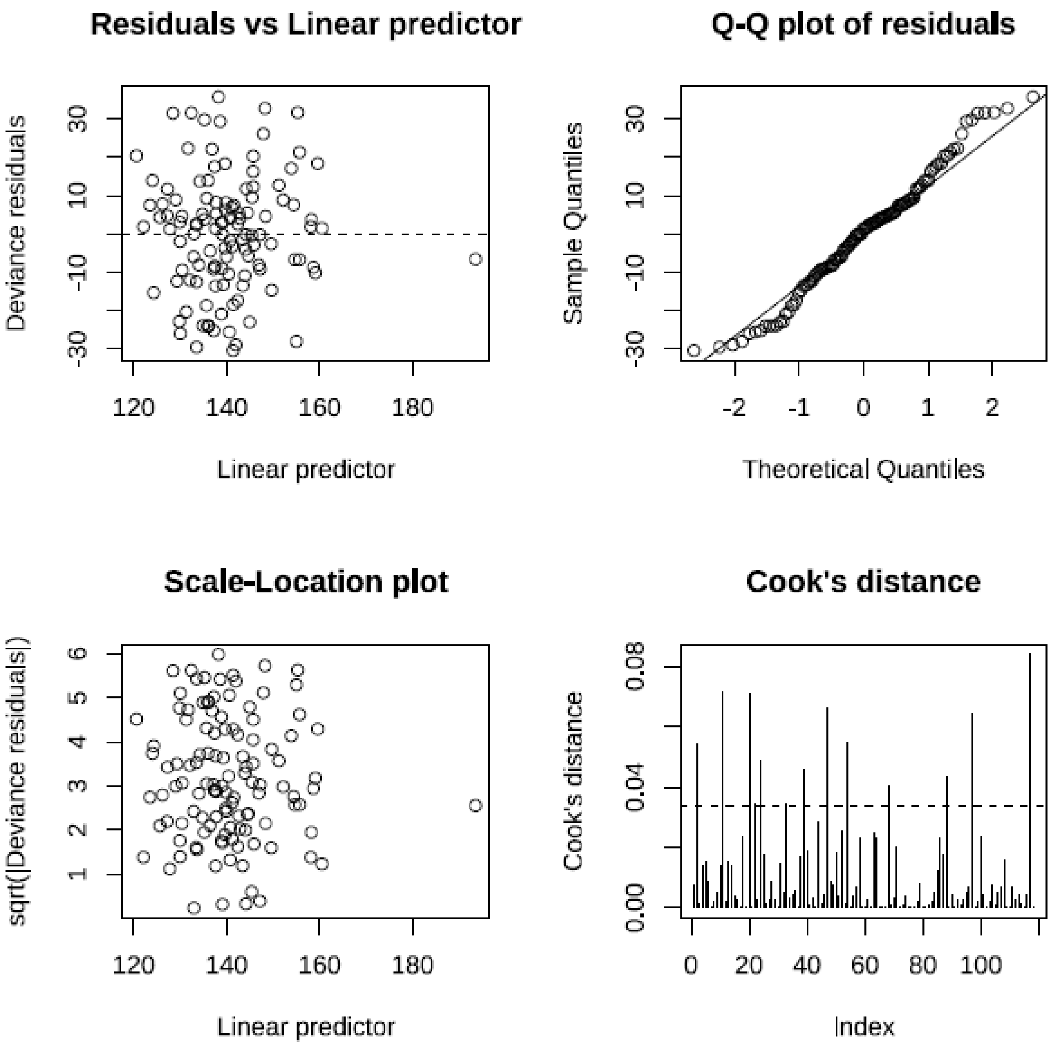

12) GAM model validation focusing on Diurnal mDBP and LDL-C

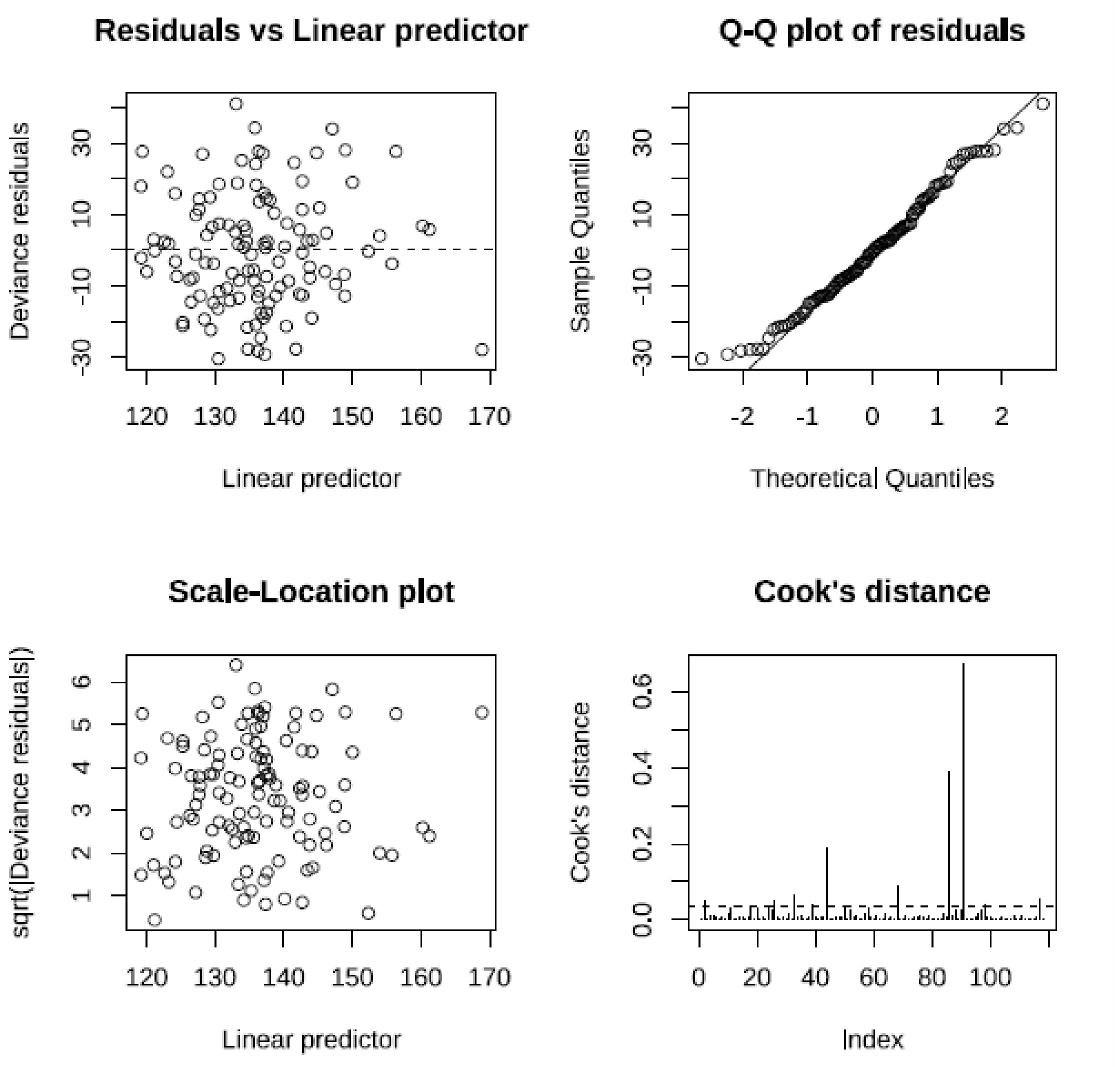

13) GAM model validation focusing on Nocturnal mSBP and LDL-C

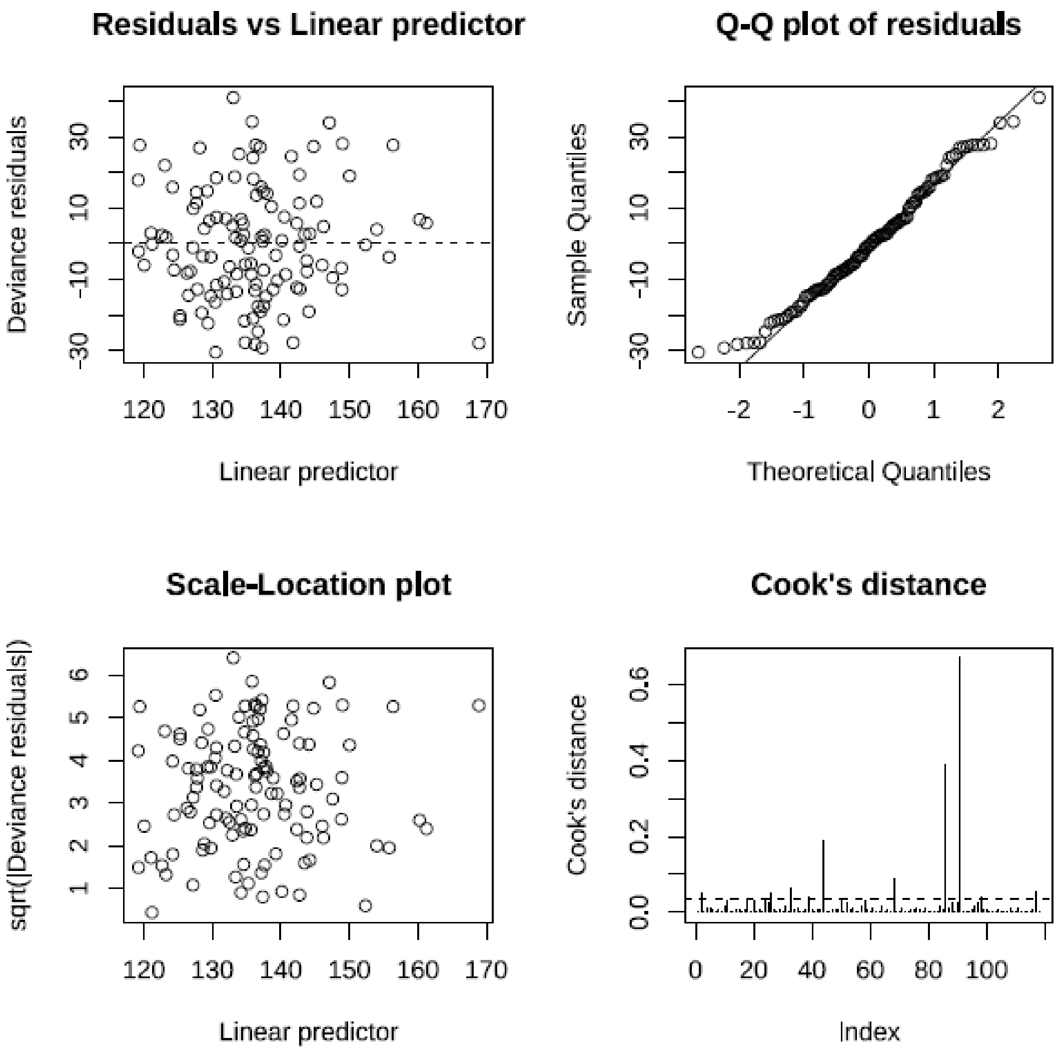

14) GAM model validation focusing on Nocturnal mDBP and LDL-C

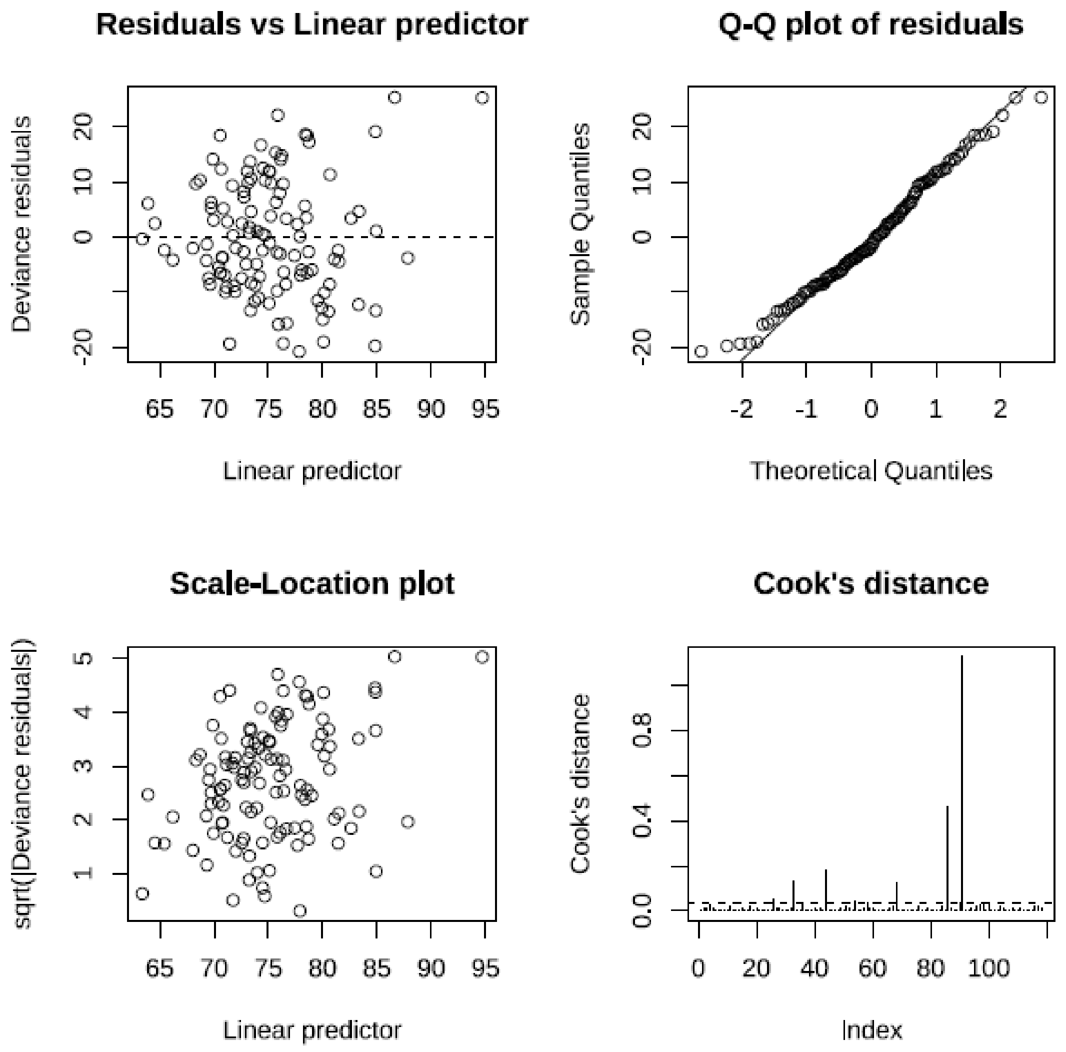

15) GAM model validation focusing on NSBPDR and LDL-C

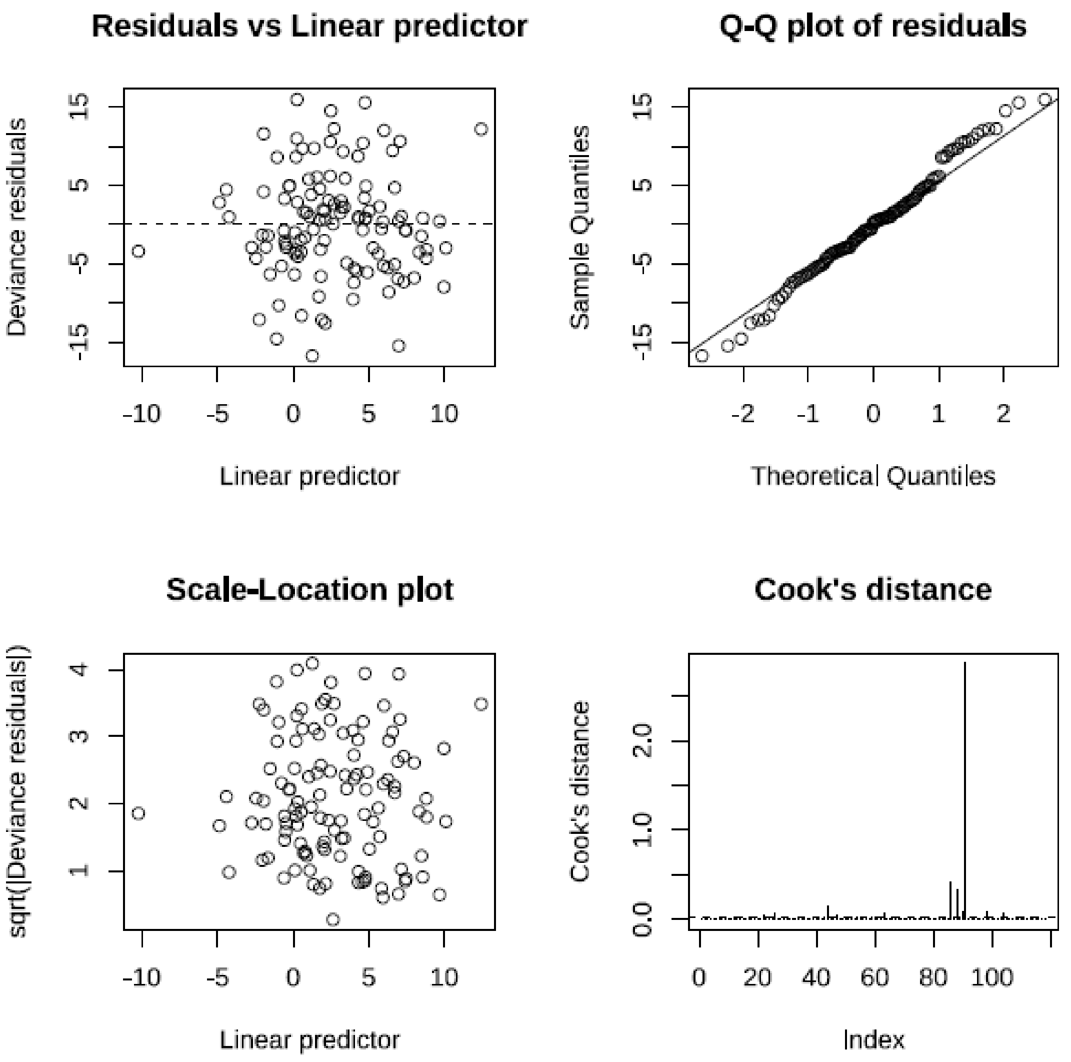

16) GAM model validation focusing on NDBPDR and LDL-C

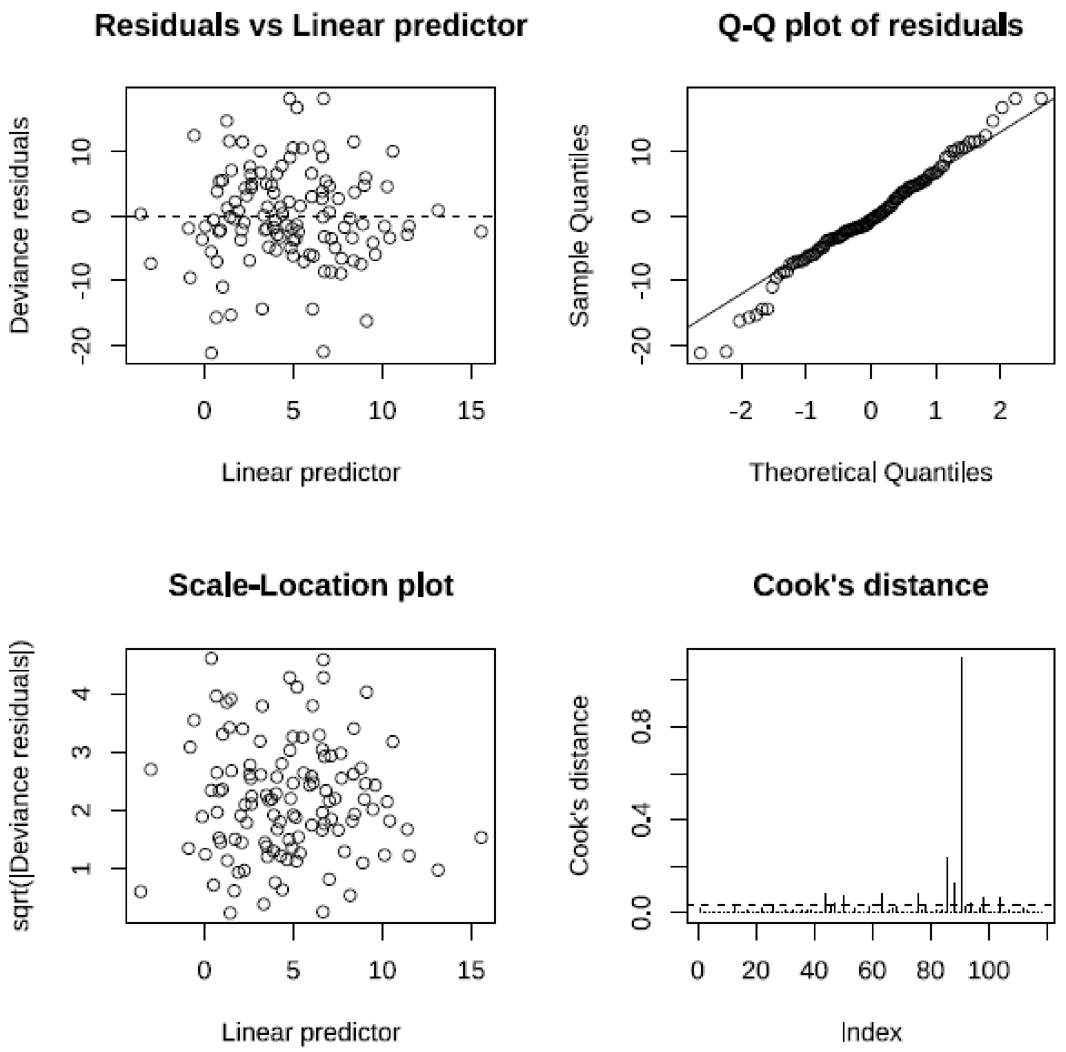

Goodness-of-fit Assessment of GAM Models between HDL-C and Blood Pressure Parameters

Model	R2	Deviance explained	GCV	AIC	BIC	Log-likelihood	n
24h-mSBP	0.1238	0.3267	372.2653	1,027.39	1,108.02	-484.60	118
24h-mDBP	0.1083	0.2989	151.8791	922.85	997.66	-434.43	118
Diurnal mSBP	0.1220	0.3256	393.1156	1,033.80	1,114.53	-487.77	118
Diurnal mDBP	0.0681	0.2672	156.4233	926.33	1,001.14	-436.17	118
Nocturnal mSBP	0.0342	0.2406	425.8313	1,044.51	1,119.32	-495.25	118
Nocturnal mDBP	-0.0387	0.1832	181.9666	944.18	1,018.99	-445.09	118
NSBPDR	0.0206	0.2299	73.4887	837.19	912.00	-391.60	118
NDBPDR	-0.0556	0.1700	90.9250	862.31	937.12	-404.16	118

GCV: Generalized Cross-Validation; AIC: Akaike Information Criterion; BIC: Bayesian Information Criterion; NSBPDR: Nocturnal systolic blood pressure decline rate; NDBPDR: Nocturnal diastolic blood pressure decline rate

17) GAM model validation focusing on 24h mSBP and HDL-C

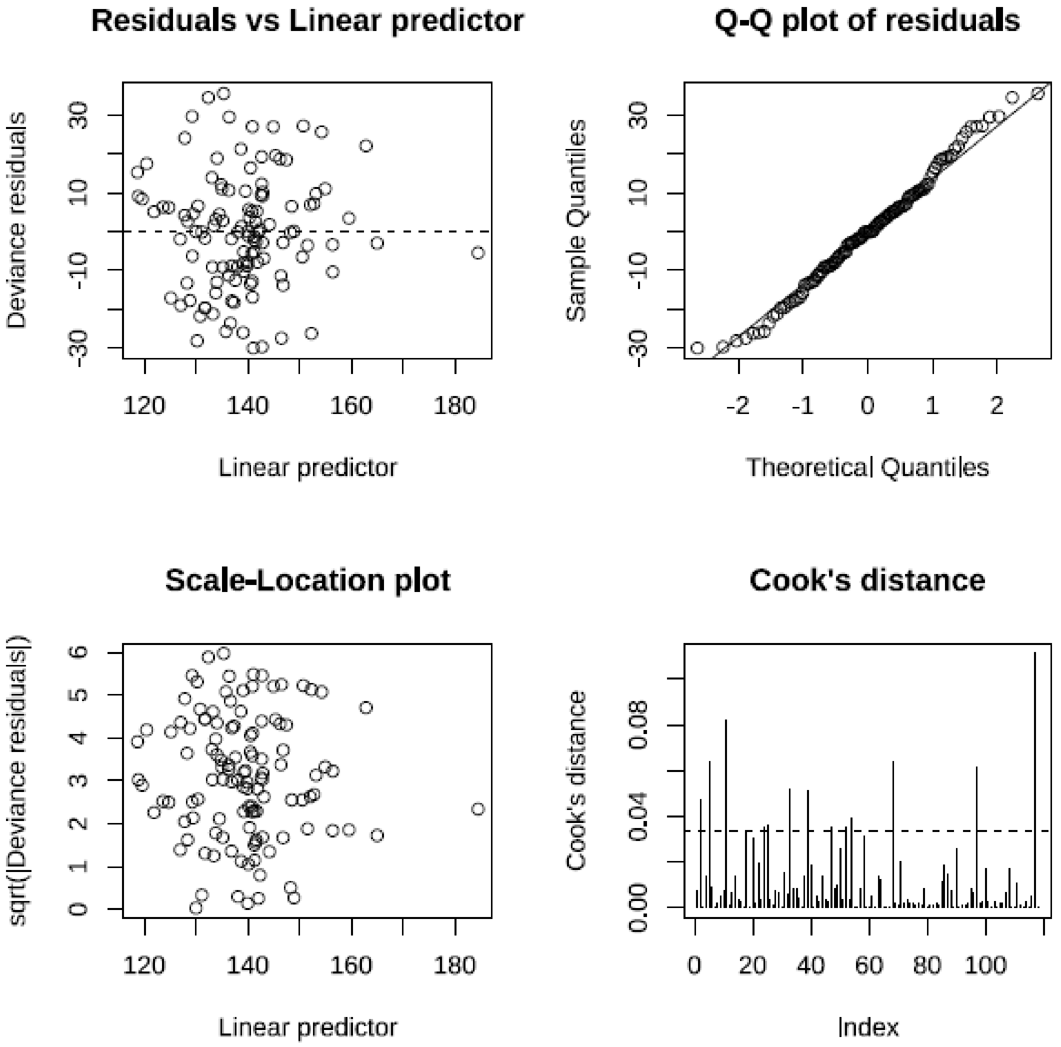

18) GAM model validation focusing on 24h mDBP and HDL-C

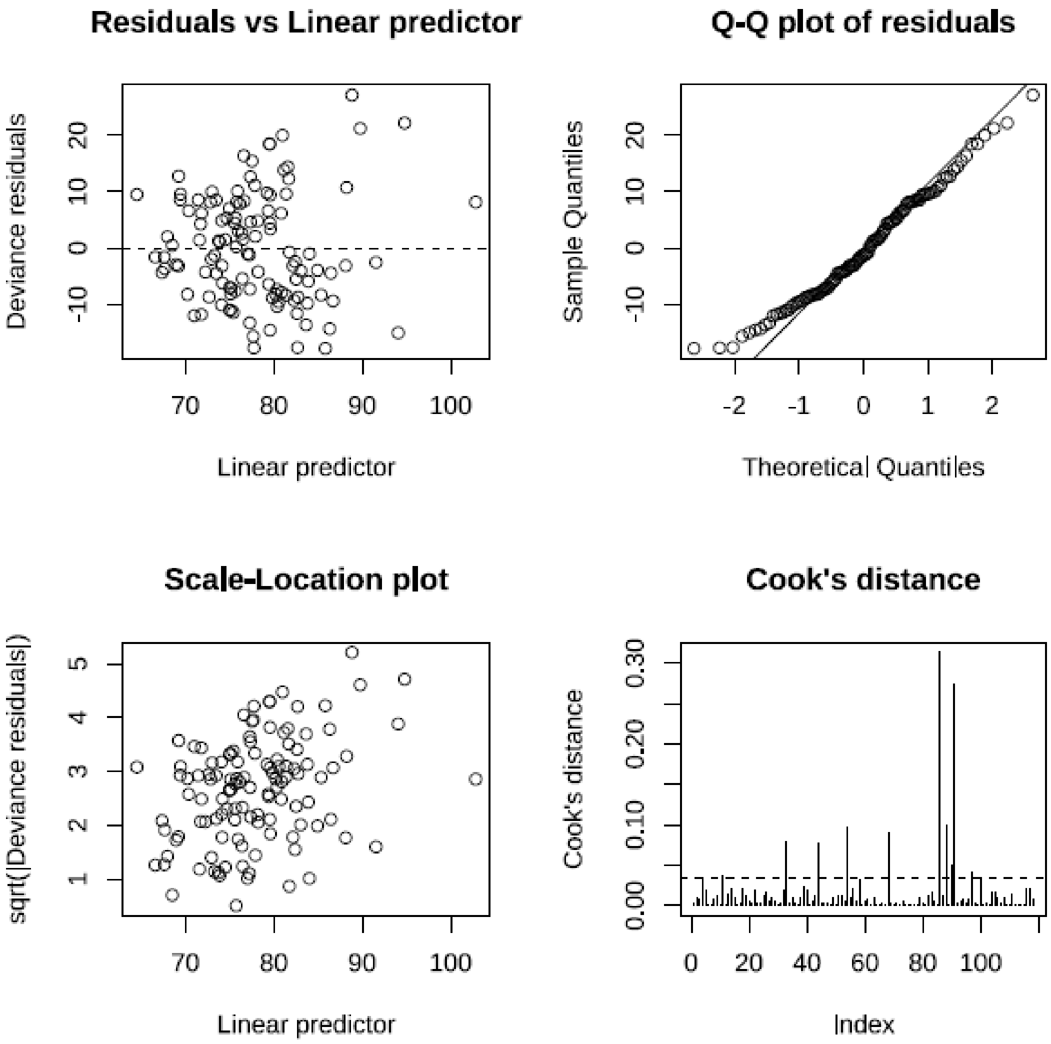

19) GAM model validation focusing on Diurnal mSBP and HDL-C

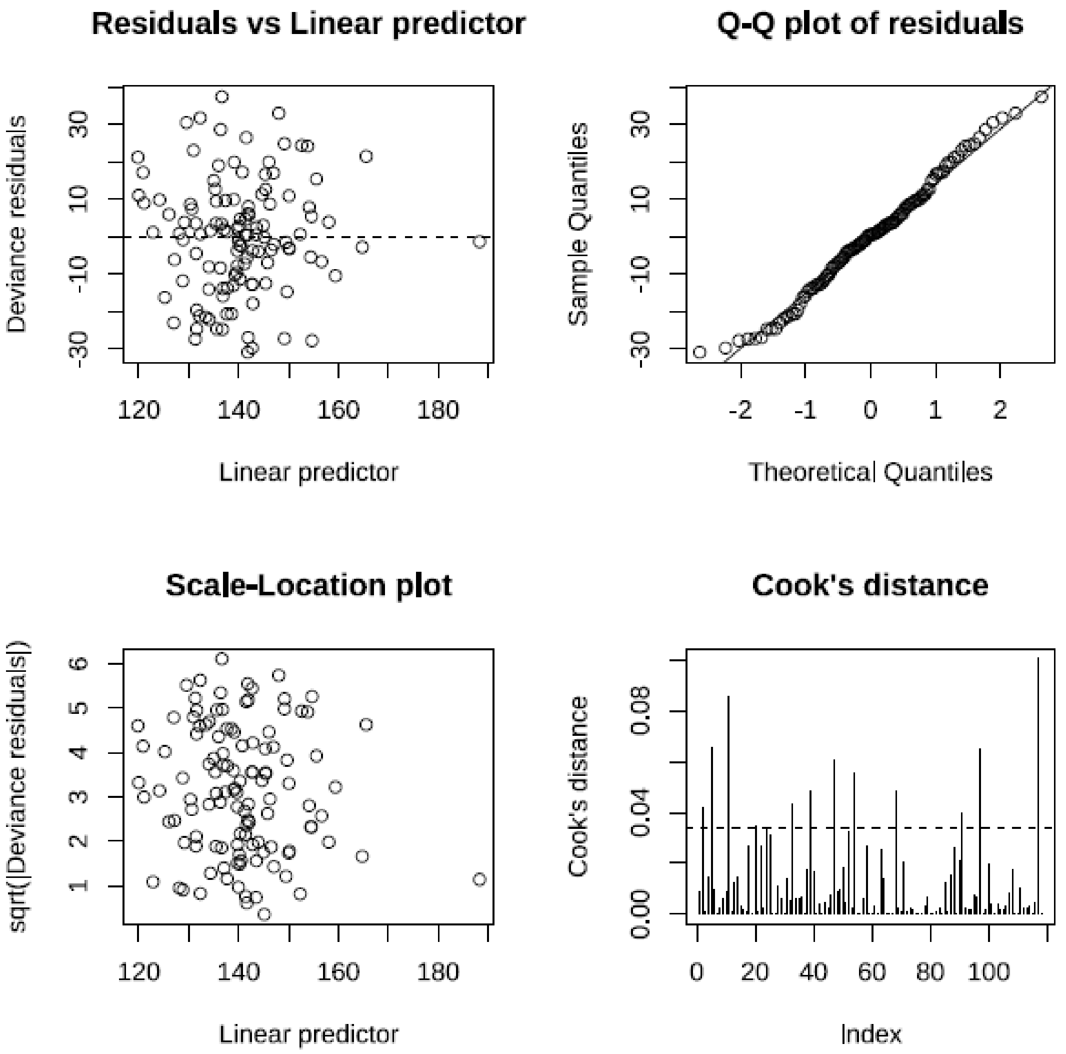

20) GAM model validation focusing on Diurnal mDBP and HDL-C

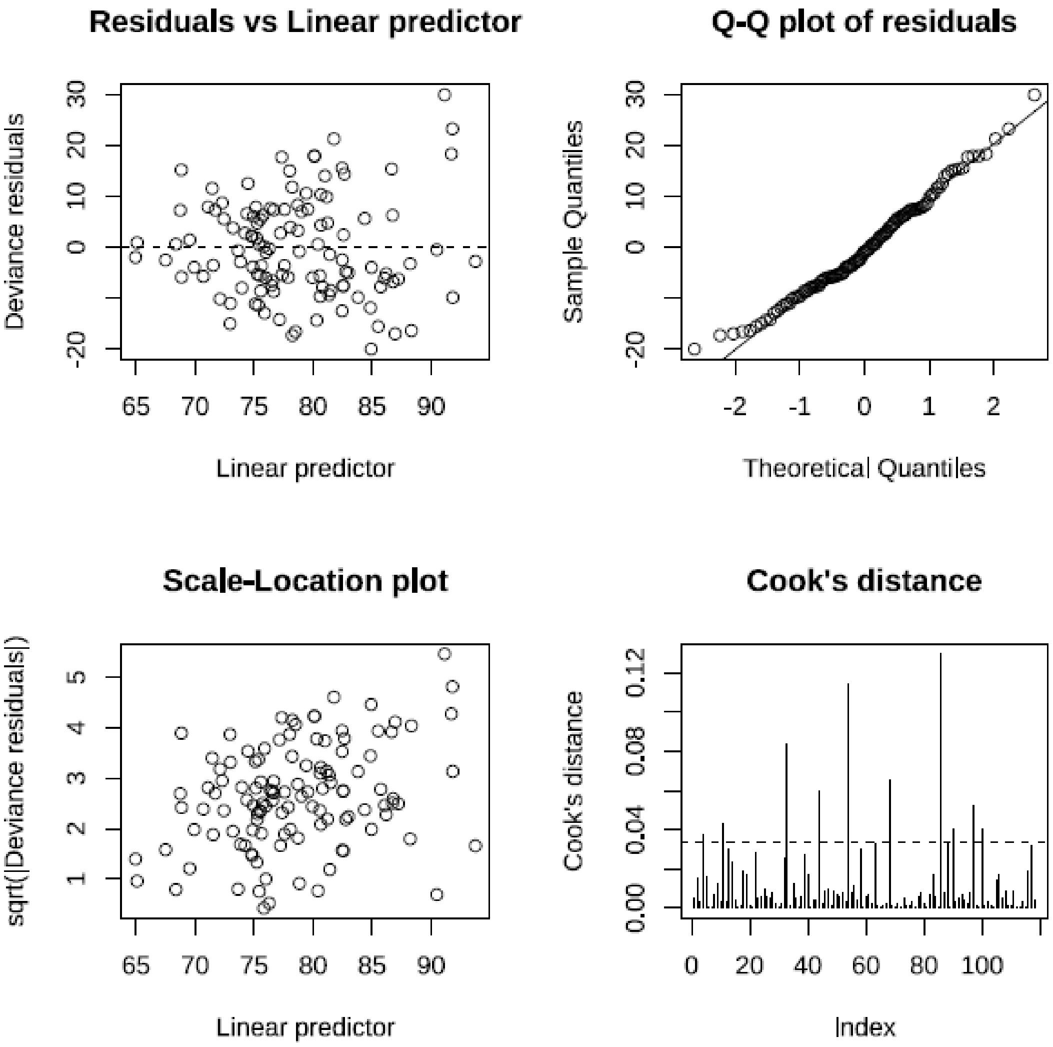

21) GAM model validation focusing on Nocturnal mSBP and HDL-C

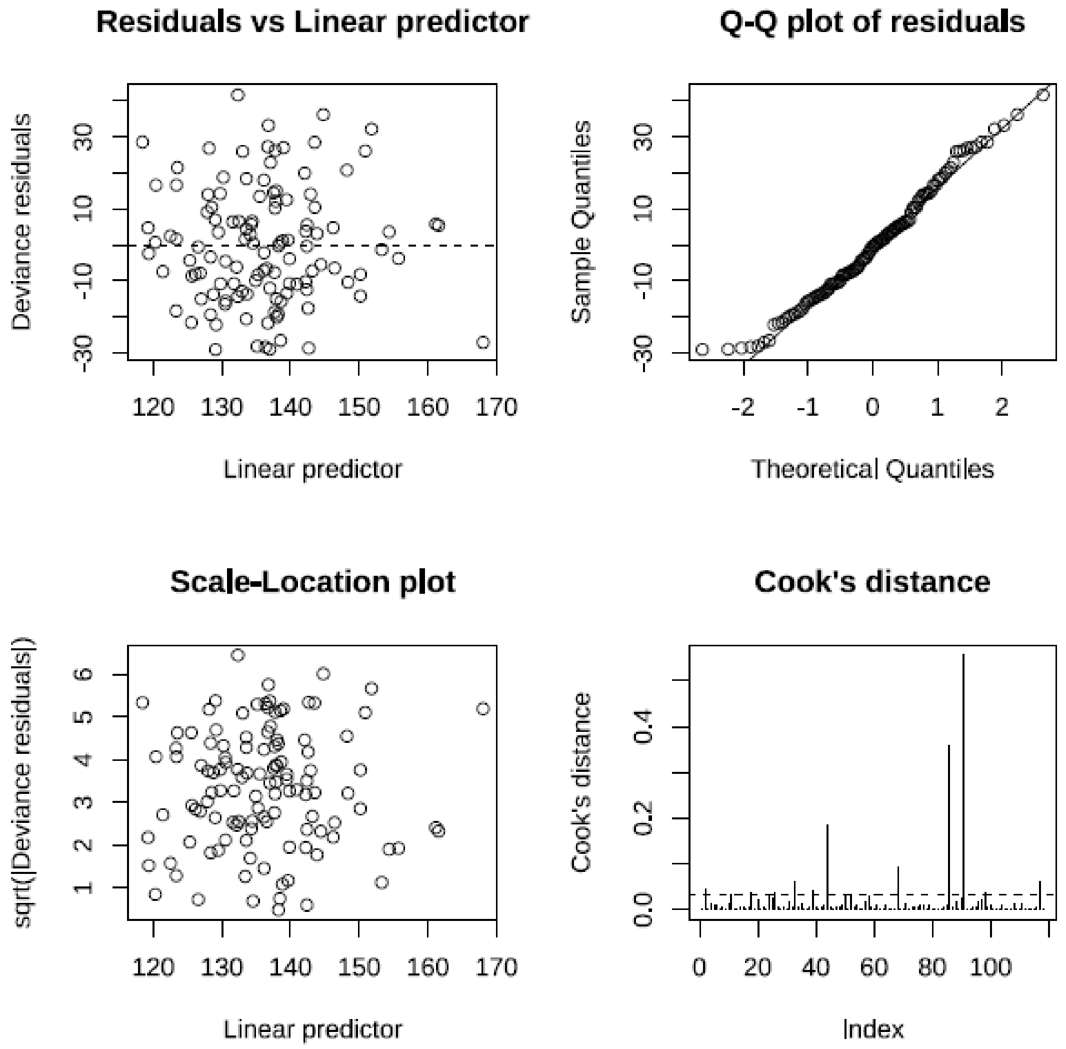

22) GAM model validation focusing on Nocturnal mDBP and HDL-C

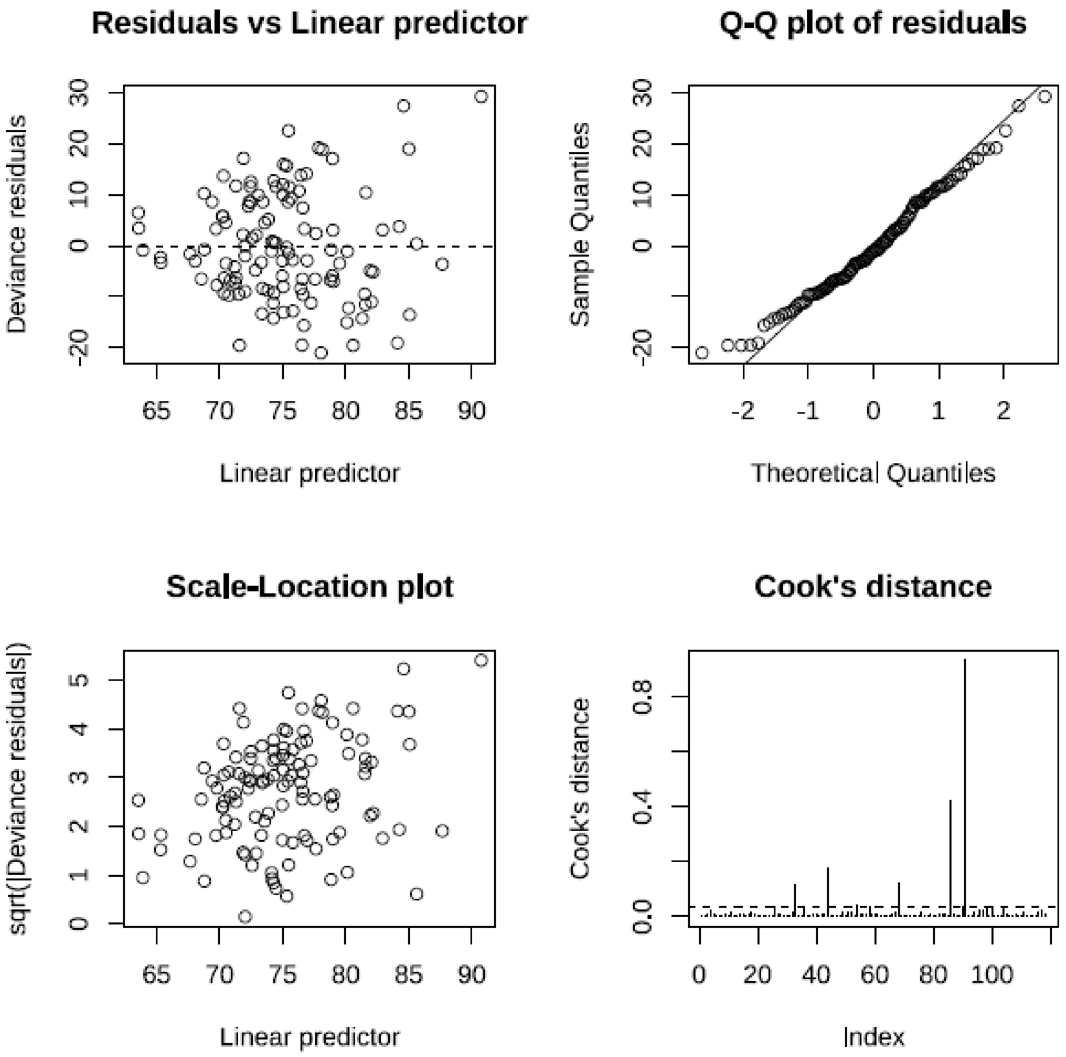

23) GAM model validation focusing on NSBPDR and HDL-C

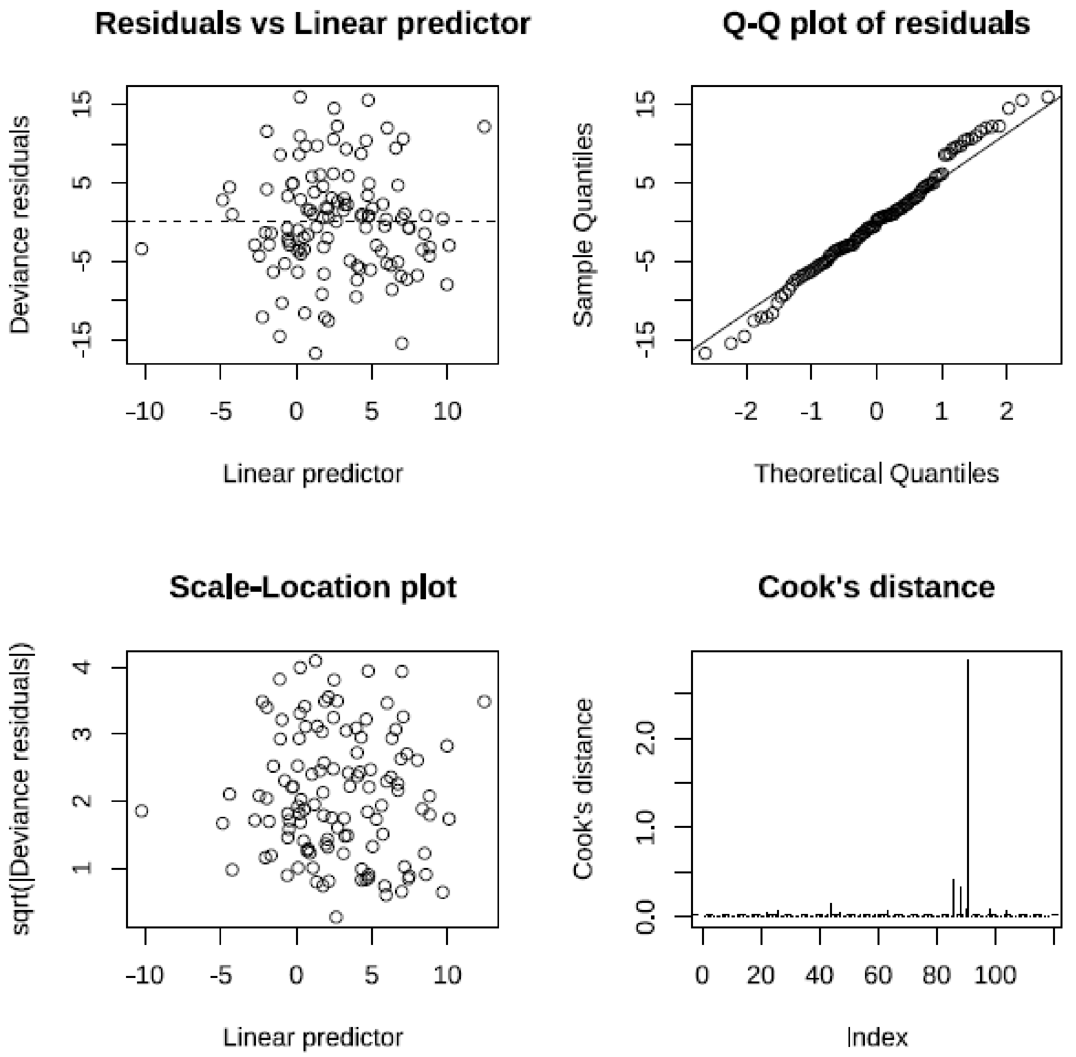

24) GAM model validation focusing on NDBPDR and HDL-C

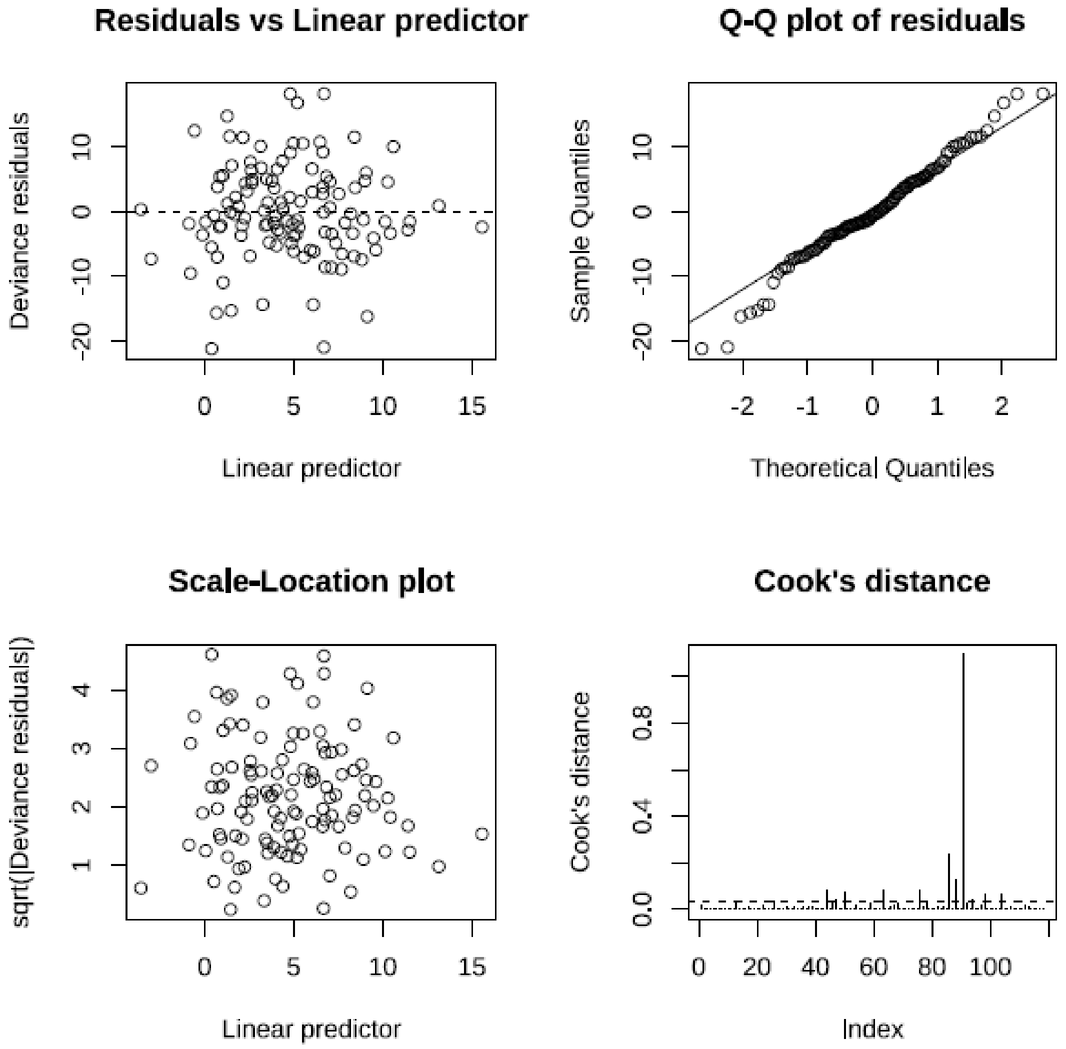

PS: df：Degrees of Freedom; AIC：Akaike Information Criterion; BIC：Bayesian Information Criterion; Residual df：Residual Degrees of Freedom; NSBPR：Nocturnal Systolic Blood Pressure Diurnal Rate; NDBPR：Nocturnal Diastolic Blood Pressure Diurnal Rate; Obs.：Observations
